# GeLC-MS-based proteomics of *Chromobacterium violaceum*: comparison of proteome changes elicited by hydrogen peroxide

**DOI:** 10.1038/srep28174

**Published:** 2016-06-20

**Authors:** D. C. Lima, F. T. Duarte, V. K. S. Medeiros, P. C. Carvalho, F. C. S. Nogueira, G. D. T. Araujo, G. B. Domont, S. R. Batistuzzo de Medeiros

**Affiliations:** 1 Laboratório de Biologia Molecular e Genômica, Centro de Biociências, Universidade Federal do Rio Grande do Norte, Campus Universitário s/n, Lagoa Nova, Natal, RN, 59072-970 Brazil; 2 Instituto Federal de Educação, Ciência e Tecnologia do Rio Grande do Norte (IFRN), RN, Brazil; 3 Colegiado de Medicina, Universidade Federal do Vale do São Francisco, Avenida José de Sá Maniçoba, S/N, Centro, Petrolina, PE, 56304-917, Brazil; 4 Laboratório de Proteômica e Engenharia de Proteínas, Instituto Carlos Chagas, Fiocruz, PR, Brazil; 5 Unidade Proteômica, Laboratório de Química de Proteínas, Bloco A - sala 543, Centro de Tecnologia, Universidade Federal do Rio de Janeiro, Av. Athos da Silveira Ramos, 149, Cidade Universitária, Rio de Janeiro, RJ, 21941 – 909, Brazil

## Abstract

*Chromobacterium violaceum* is a free-living bacillus with several genes that enables it survival under different harsh environments such as oxidative and temperature stresses. Here we performed a label-free quantitative proteomic study to unravel the molecular mechanisms that enable *C. violaceum* to survive oxidative stress. To achieve this, total proteins extracted from control and *C. violaceum* cultures exposed during two hours with 8 mM hydrogen peroxide were analyzed using GeLC-MS proteomics. Analysis revealed that under the stress condition, the bacterium expressed proteins that protected it from the damage caused by reactive oxygen condition and decreasing the abundance of proteins responsible for bacterial growth and catabolism. GeLC-MS proteomics analysis provided an overview of the metabolic pathways involved in the response of *C. violaceum* to oxidative stress ultimately aggregating knowledge of the response of this organism to environmental stress. This study identified approximately 1500 proteins, generating the largest proteomic coverage of *C. violaceum* so far. We also detected proteins with unknown function that we hypothesize to be part of new mechanisms related to oxidative stress defense. Finally, we identified the mechanism of clustered regularly interspaced short palindromic repeats (CRISPR), which has not yet been reported for this organism.

*Chromobacterium violaceum* is a free-living Gram-negative bacillus, an opportunistic pathogen that inhabits the soil and waters of the Negro River in the Brazilian Amazon^1^. It is known for its abilities to produce cyanide^2^, solubilize gold^3^, produce chitinolytic enzymes^4^ and synthesize bioplastics^5^. *C. violaceum* has several biotechnological applications such as bioremediation^6^, production of cellulose^7^ and compounds acting as anticancer agent^8^. Its primary characteristic is the production of a violet pigment, violacein, that has several pharmacological properties such as antitumoral and antimicrobial activities^9^. Due to a significant biotechnological potential, its genome was sequenced in 2003, identifying a chromosome of 4,751,080 bp containing 4,431 Open Reading Frames, 40% of them coding for hypothetical proteins^10^.

Genome analysis showed that this organism contains several genes associated to environmental acclimation and stress tolerance, indicating physiological versatility in regard to environmental changes; examples of such genes are: *rpoS*, LysR, AraC and TetR families^11^.

Proteomics enables investigating how organisms acclimatize to harsh environmental conditions, as the proteome, differently the genome, is dynamic. The first proteomic studies of *C. violaceum* investigated the effects of cyanide on the bacterial metabolism^12^ while Ciprandi and co-workers evaluated the response to arsenic stress in this organism^13^. Cordeiro and colleagues analyzed how different growth temperatures influences *C. violaceum* metabolism while Lima *et al*. performed a GeLC-MS proteomics approach in the presence of high concentration of iron^14^,^15^. However, previous proteomic profilings disclosed at most, about 400 proteins from *C. violaceum* in response to an environmental stress.

In bacteria, several environmental factors, such as acid exposure, high salt concentrations and treatment with antimicrobial compounds can induce changes in the redox state of the cellular environment and lead to oxidative stress^16^. The intense selective pressure imposed by oxidative stress has resulted in several detoxification and defense pathways^17^. *C. violaceum* is subject to these pressures due to its lifestyle, where it is constantly exposed to external biotic and abiotic variations^11^. As such, this organism possesses a robust defense mechanisms to cope with environmental challenges. Exposure to hydrogen peroxide and other reactive oxygen species (ROS) is a universal characteristic of life in aerobic environment^18^. Hydrogen peroxide induces oxidative stress. It can accumulate to significant levels within the cell and is a widely used chemical model in experimental studies for oxidative stress. It is produced endogenously within organisms as a byproduct of oxidative phosphorylation and other reactions. In mammals, plants and even bacteria, it is produced in elevated levels as a form of defense against pathogens^19^,^20^.

Many transcriptome and proteome studies used hydrogen peroxide as a model to analyze oxidative stress^21^,^22^,^23^,^24^,^25^,^26^,^27^. These investigations detected changes in the expression pattern of several genes such as sigma factors and MerR family which are involved in general stress response. They also found that exposure to hydrogen peroxide induces the gene expression and mechanisms associated with oxidative stress, such as genes that encode catalase, thioredoxin reductase and peroxidase as well as mechanisms of iron and manganese homeostasis and SOS response.

High peroxide levels in the cellular environment can damage proteins with prosthetic groups of iron, releasing this metal to the cellular environment. Iron, in turn, reacts with hydrogen peroxide through the Fenton reaction, forming highly reactive species such as hydroxyl radical (•OH)^17^, which oxidizes biomolecules including lipids, DNA and proteins^19^.

The present study investigates changes in the proteome profile under oxidative stress induced by a high dose of hydrogen peroxide; we believe these changes reflect the mechanisms used by this bacterium to cope with the oxidative stress. Moreover, our results provided experimental evidence on hypothetical proteins and thus shortlisting the approximately 40% of the ORFs annotated as hypothetical proteins. Here, we used an LTQ-Orbitrap *Velos* to obtain a substantial comprehensive proteomic profile of an organism that is protecting itself against ROS. Given the nature of the experiment at hand, these proteins are highly likely to be related to oxidative stress defense.

## Results and Discussion

### Temporal changes of physiological and metabolic activities in *C. violaceum* cells in response to treatment with H_2_O_2_

**Figure 1:**
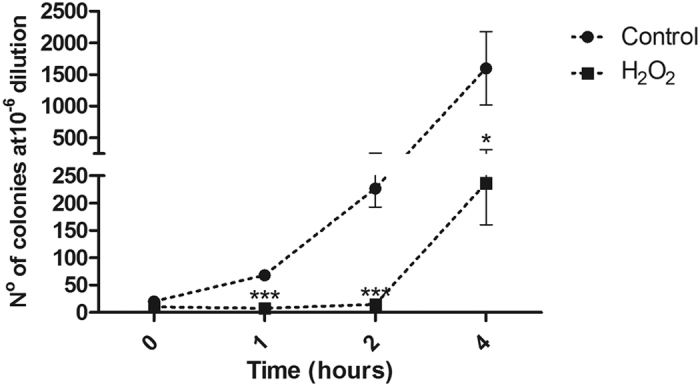
CFU count of *C. violaceum* exposed to 8 mM H_2_O_2_. During treatment, bacteria cells did not grow, although were not dead. After two hours of growth, *C. violaceum* starts to recover from the oxidative stress exposure. Each point of the curve represents the mean from three biological replicates, each one plated twice. *p < 0.05, ***p < 0.001 (T-test for comparisons between negative control on its respective time).

Two growth curves were made to investigate how *C. violaceum* behaves under oxidative stress induced by 8 mM of H_2_O_2,_: the first curve relied on the colony-forming unity (CFU) count and the second was traced according to the optical density (OD) measurement at 600 nm (Fig. 1 and Supplementary Fig. S1, respectively). *E. coli* CC104 was used as reference organism for data comparison; however, this dose proved to be lethal to this organism, making the comparison impossible. This suggests that *C. violaceum* displays far more efficient mechanisms of peroxide detoxification and oxidative stress tolerance than those of *E. coli.* Both growth curves (OD measurement and CFU count) show that *C. violaceum* tolerates the environment containing H_2_O_2_ in the first hour of treatment, although no population growth was observed. This only occurs from the second hour onwards (Fig. 1 and Fig. S1). In a microarray assay of *Desulfovibrio vulgaris* treated with the same agent, the authors found that the greatest variation in gene expression occurred in two hours^28^. This indicates that from this time onwards the cell may express a substantial number of genes as a defense mechanism against oxidative stress. Based on this result, the authors defined a time of 2 hours to analyze the proteomic profile. Kim and colleagues also carried out a proteome study using hydrogen peroxide as a stress-inducing agent in *Salmonella enterica*, with a treatment time of 120 minutes^22^. Thus, we established a time period of 2 hours to conduct the differential proteome assay in *C. violaceum*. The 8 mM hydrogen peroxide concentration was defined by 1D-SDS-PAGE gel analysis. To this end, bacteria were cultivated under the conditions previously described and with increasing concentrations of hydrogen peroxide; 2, 4, 8, 16, and 32 mM. Under these conditions, we verified major visual changes in the 1D-SDS-PAGE band patterns at the 8 mM concentration (Supplementary Fig. S2).

**Figure 2:**
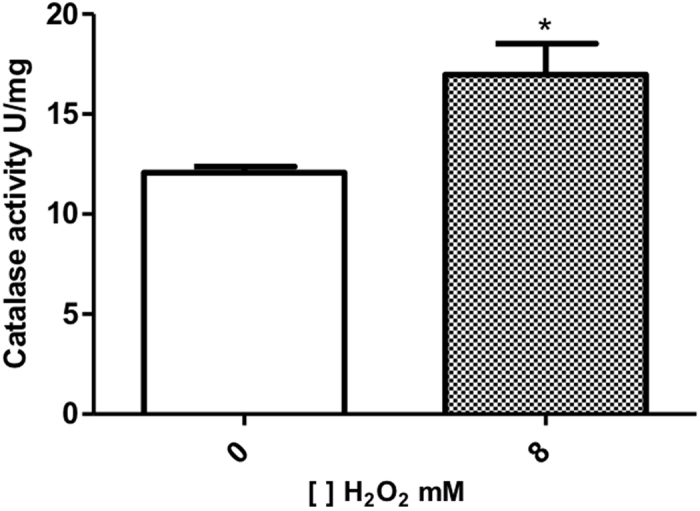
Catalase activity in the *C. violaceum*. Each column represents the mean value ± SD of 3 separate experiments, p < 0.05. (column 1: control group; column 2: bacteria cultured with 8 mM of H_2_O_2_).

To determine if the 8 mM H_2_O_2_ used to treat *C. violaceum* was able to induce oxidative stress, the catalase activity was measured. The result disclosed in Fig. 2 show a significant increase on enzymatic activity, as expected when bacteria is under oxidative stress^29^. This observation is relevant as an indicator of oxidative status for proteomic analysis. Although the TFold analysis, which pinpoints constitutive proteins with differential abundance^30^, did not point as significant the increase in Kat (catalase) expression, this upregulation may be enough to lead to an increase in catalase activity.

### Proteomics analysis in *C. violaceum*

Proteomics techniques were applied to investigate the response of *C. violaceum* under oxidative stress, using 8 mM of H_2_O_2_ as stress inducer. Three biological replicates and two technical replicates were used for this analysis. The Venn diagram shows that 1,483 proteins were identified in our survey; from these, 1,175 were found in both conditions, 131 in the control and 177 only in the 8 mM H_2_O_2_ condition (Supplementary Fig. S3). Hereafter, we refer to exclusively identified proteins as those identified in at least two biological replicates of one condition and in no replicates of the other. The literature has shown, in general, a weak correlation between the abundance of gene transcripts and protein^31^,^32^. Notwithstanding this, we further investigate if this postulation holds true for Ferredoxin and BioC. The motivation for shortlisting these genes/proteins is in selecting an example of a protein with decreased abundance after the hydrogen peroxide treatment and another being exclusively identified in the control condition, respectively. Quantitative PCR revealed a positive correlation between the mRNA abundance of the ferredoxin and *bioC* genes and their corresponding proteins (Supplementary Fig. S4).

The 30 proteins with most spectral counts are listed in Table 1. These results showed that the most abundant proteins in the proteome of *C. violaceum* exhibit constitutive expression and 83% are associated with general metabolism. Anderson and colleagues assessed protein abundance in the proteome of the cyanobacterium *Nostoc punctiforme* and reported the majority of the most abundant proteins to belong to general metabolism^33^.

### Functional classification of proteins

**Figure 3:**
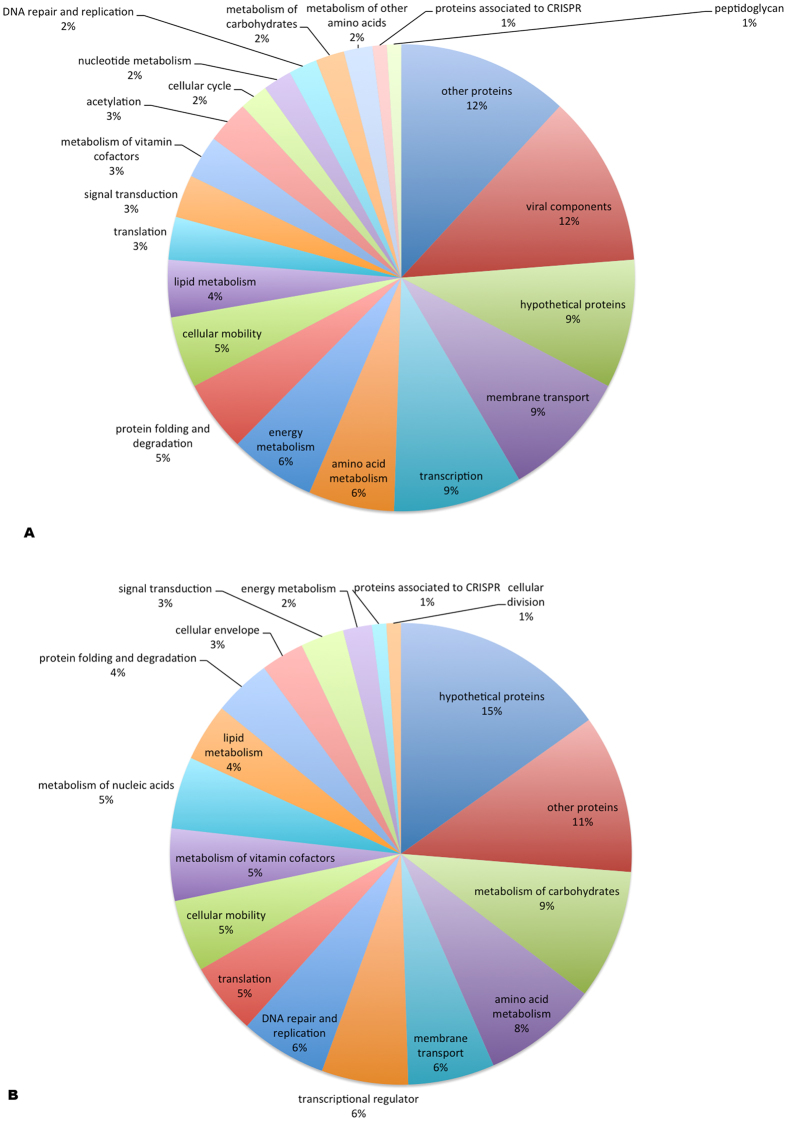
Functional classification of proteins. (**A**) Exclusively identified proteins from oxidative stress condition. (**B**) Proteins identified exclusively in control condition.

The functional classification of the identified proteins was generated using DAVID^34^. Figure 3A shows the categories of proteins exclusively identified in the treatment condition; likewise, 3B for those exclusive to the control. Among the proteins in group 3A we highlight several viral components and CRISPR proteins that are discussed below.

The category of hypothetical proteins was the most representative in the control condition. Proteins with unknown function are very common in prokaryotic genomes indicating that much of the genetic patrimony is still unknown. So, they represent a very interesting class to be further analyzed and characterized, in order to identify new genes with biotechnological potential yet unidentified. The others proteins expressed in control condition are for housekeeping. Probably, there is a decreasing in the abundance of these proteins to prioritize the production of proteins related to a better acclimation strategy to endure oxidative stress^35^,^36^.

### Re-annotation of expressed hypothetical proteins

Among the proteins identified exclusively under control and oxidative stress conditions, 71 are characterized as hypothetical ORFs and/or conserved hypothetical, of which 40 are expressed only in treatment and 31 in control conditions. However, new analyses conducted with BLASTp tool indicated that 36 ORFs display homology with proteins already characterized (Supplementary Tables S2 and S3) demonstrating the importance of genome re-annotation to optimize research performed in the post-genome era. Gundogdu and colleagues re-annotated and reassessed the genome of *Campylobacter jejuni*, pointing out the importance of updating genomic data for studies of these and other organisms^37^. After re-annotation, 35 ORFs remained as proteins with unknown function: 19 for oxidative stress and 16 under control conditions. This shows that new mechanisms related to resistance and/or detoxification processes may be present in *C. violaceum* and other bacteria. Genome analysis indicates that some of these ORFs (CV_4246, CV_3909, CV_0053 and CV_3599) display operon organization. ORF CV_4246 for example, is adjacent to the *xth*A gene, which encodes for an endonuclease. As such, this ORF may be related to DNA repair, which is activated under oxidative stress.

### Proteome response of *C. violaceum* to H_2_O_2_

#### Cellular protection mechanisms against oxidative stress

The ability to detoxify reactive oxygen species is vitally important to the survival of microorganisms, particularly those subjected to sudden environmental changes such as free-living species^38^. As far as we know, this is the first study that analyzes the response of *C. violaceum* to an extreme oxidative stress. Nevertheless, we recognize that a previous report from Lima and colleagues investigated the proteome response of *C. violaceum* in the presence of iron, which leads, to some extent, to oxidative stress^15^. Stress generally induces the expression of proteins capable of protecting cells from oxidative damage^39^. Organisms typically respond to oxidative stress via several defense processes against the stressor: (1) inactivation of the stressor (e.g.: AhpC, Catalase and Superoxide Dismutase); (2) protecting macromolecules against oxidative inactivation (e.g.: Dps and MrgA); (3) repairing damaged molecules (TrxA) and (4) degrading irreversibly- damaged proteins (e.g.: proteases)^40^. These examples establish a classic response to oxidative stress. However, in the present study only glutathione s-transferase (Gts and Gts) and proteases (ColA, PepP, Pcp, PtrB and CV_3452) were detected in the hydrogen peroxide treated samples (Supplementary Table S4). The remaining proteins are present in the genome of *C. violaceum*, but showed no significant alteration in abundance (Supplementary Table S1). The low variation in expression may occur because the behavior of *C. violaceum* is similar to that of *Neisseria gonorrhoeae*, which exhibit 100 times more catalase activity than *E. coli*^41^. Thus, the elevated baseline production of catalase in this bacterium may provide greater resistance to hydrogen peroxide and activation becomes insignificant under stress.

Glutathione s-transferase works to protect against a range of environmental stressors caused by hydrogen peroxide, including oxidative stress^42^. Two glutiathiones s-transferase (Gst and Gst3) are evident in the proteome of *C. violaceum* under oxidative stress (Supplementary Table S4). The versatility of this protein against stress is also observed in proteomic analysis of *Enterococcus faecalis V583*, where it occurs in physiological response to stress provoked by bile^43^.

Proteins involved in folding and degradation represent 5% of proteins expressed after treatment (Fig. 3A). The identification of 5 proteases (ColA, PepP, Pcp, PtrB and CV_3452) indicates the presence of a proteolytic pathway that functions as a secondary antioxidant defense mechanism, degrading damaged proteins, a mechanism that was independently proposed by Davies and Stadtman in 1986 as mentioned in Cabiscol and Ros^44^. Chaperones present in differential protein analyses after oxidative stress induction, is reported for other organism such as *Fusobacterium nucleatum*^45^ and *Staphylococcus aureus*^46^. These proteins may play a role in the protection and refolding of proteins oxidized by hydrogen peroxide.

#### DNA repair

Oxidative stress is one of the main causes of DNA damage. After treating *C. violaceum* with hydrogen peroxide, an endonuclease (Nth) and two glycosylases (MutM and Udg) were exclusively identified (Supplementary Table S4). These genes are components of the base excision DNA repair pathway, the main mechanism used by living cells to repair oxidative lesions^47^. Induction of DNA repair genes by hydrogen peroxide in the proteome of *Desulfovibrio vulgaris Hildenborough* was found by Gaucher and colleagues^48^. However, their analysis did not detect genes from the BER pathway, whose role is highly significant in removing these oxidative lesions, enabling the organism to survive under conditions of stress. DinB that belongs to the SOS regulon was also identified in our work under oxidative stress. The SOS response in bacteria includes an overall physiological response by inducing the transcription of several genes in response to DNA damage^49^. However, the key gene in this response is not present in the genome of *C. violaceum*^50^ and as such, it may be activated by another mechanism. Recently, Schook *et al*. described a SOS-like mechanism in *Neisseria gonorrhoeae*, inducible by oxidative stress^51^. *C. violaceum* may have such a mechanism, since the orthologous gene that encodes the regulatory protein of this SOS is present in its genome. Organisms from the *Bacillus* genus appear to display only non-significant induction of genes belonging to the SOS regulon under peroxide-induced stress^52^.

#### Modulators of gene expression

Bacteria exposed to hydrogen peroxide respond by expressing enzymes to detoxify and repair the damage. This response is generally regulated by redox-sensitive transcription factors^18^. Under oxidative stress, proteins that control baseline transcription mechanisms emerge, such as sigma factors s^32^ (RpoH), s^38^ (RpoS) (Supplementary Table S4). A wide range of transcription regulators were also expressed, which react with alternative sigma factors involved in bacterial cell response to stress, such as those belonging to AraC, TetR, MarR and Mer (Supplementary Table S4). Two members of the AraC family of transcriptional regulators can be found in the proteome of *C. violaceum* under stress. AraC family is one of the largest groups of protein regulators in bacteria and is involved in regulating the transcription of a variety of cellular processes in Gram-negative and Gram-positive bacteria, including carbon metabolism in response to stress and virulence^53^. MerR family represses the expression of genes that detoxify metallic ions through efflux and sequestration of metals. In *N. gonorrhoeae*, it activates the NmlR regulon that contains the *adh*C gene (glutathione-dependent alcohol dehydrogenase). NmlR appears to defend bacterial cells against carbonyl stress and, in some cases, nitrosative stress^54^. TetR, in turn, represses efflux multidrug transporter genes^55^.

A total of 3% of proteins exclusively identified in oxidative stress-responsive proteome are involved in acetylation (e.g.: CV_0453, CV_3388 and CV_3155, all of them are uncharacterized proteins with acetyltransferase domains), associated to post-translational modifications (PTMs) (Supplementary Table S4). Many PTMs play a critical role in regulating protein levels in bacteria^56^. Acetylation is characterized by regulation of enzyme function and participation in signal transduction^48^. In conjunction with proteins related to signal transduction, acetylations found in the proteome of *C. violaceum* likely act by modeling the gene expression response to induction by hydrogen peroxide. Proteins responsible for signal transduction (e.g.:CV_3501 and CV_0181) also account for 3% of this proteome (Supplementary Table S4). Hungria and colleagues studied *C. violaceum* genome and observed these modulators of stress response, suggesting that they participate in rapid acclimation to environmental changes endured by this organism. Expression of these proteins under hydrogen peroxide-induced stress reinforces the hypothesis raised by these authors^11^.

#### Cell motility

The expression of proteins related to flagellum biosynthesis (FlgA, FliC2 and FlhF) and chemotaxis (e.g.: Trg, and Tar) was detected in the oxidative stress condition (Supplementary Table S4). Cell motility and chemotactic ability are compromised by oxidative stress^57^. Activation of the expression of these genes under such stress restores the phenotype to the organism, conferring a characteristic that allows it to migrate in the environment in adverse conditions.

#### Pilus biogenesis and impact in the virulence

Two proteins identified (PilO and PilN), induced by hydrogen peroxide, comprise the pil operon (Supplementary Table S4). Recent studies that report the presence of this operon provide greater virulence to pathogenic bacteria such as *Xylella fastidiosa*^58^ and animal pathogens such as *Salmonela Typhi*^59^. The above studies indicate that the action of this operon favors adherence to the host, ensuring improved virulence. However, greater expression under oxidative stress indicates that, in addition to adherence, these proteins may also play a role in stress response.

#### General metabolism

The proteome of *Fusobacterium nucleatum* induced by hydrogen peroxide revealed an increase in the intracellular concentration of glycolytic enzymes and reduced ATP production^45^. The differential proteome of *C. violaceum* showed a 4.1 fold increase in abundance of the GlpD protein (Table 2). In the group of proteins exclusively identified under oxidative stress, we found proteins that belong to energy metabolism (AdhC and CV_1452, GraD; UbiB and Gph) (Supplementary Table S4). The presence of these proteins indicates the need for greater energy demand to resist damage caused by oxidative stress.

In *E. coli* steady-state, ribosomal hibernation is observed, where ribosome structure is modulated through protein binding, which induces the formation of dimers, protecting the ribosome^60^. The proteome of *C. violaceum* displays a change in the expression of genes that encode ribosomal proteins, demonstrating one configuration for the control (RpsI, RplK and RplU) (Supplementary Table S5) and another for treated condition (RplS RpmB and RimO) (Supplementary Table S4). Thus, oxidative stress likely models ribosomal structure, not only through interaction with other proteins, but also by expressing genes that improve the efficiency of translation under oxidative stress.

#### Prophage expression

De Almeida and colleagues detected the presence of 4 prophages in the genome of *C. violaceum*, which they called CVP1 to CVP4^61^. In our study, 20 of the exclusively identified proteins that emerged after exposure to hydrogen peroxide are products of ORFs present in CVP1 and CVP2 (Supplementary Table S4). The induction of prophage by hydrogen peroxide was previously reported by Loś *et al*. when investigating *E. coli*^62^. Glinkowska and colleagues exposed *E. coli* deficient strains in the *oxyR* gene to oxidative stress and observed an increase in gene expression for the λ prophage^63^. The present study indicated that this increased expression is due to several factors; for instance, hydrogen peroxide acts on the DNA molecule and reduces supercoiling, thereby exposing viral promoters, among others. Expression of phage genes may be related to pathogenicity and virulence in some *E. coli* strains^64^. In *C. violaceum*, an opportunistic pathogen, the prophage induction might also be associated with its pathogenicity, but little is known. When *C. violaceum* infects mammalian hosts, this induction may be occurring because the immunological response of neutrophils and macrophages is mediated by substantial ROS production^65^.

#### Proteins from CRISPR mechanism

**Figure 4:**
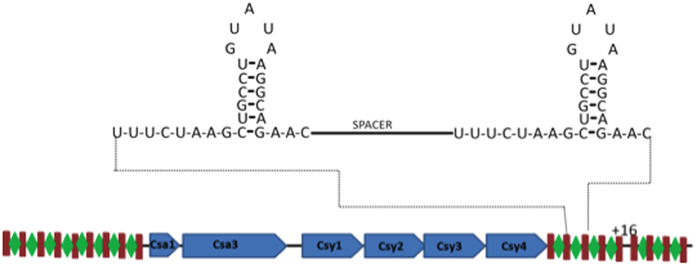
CRISPR System of *C. violaceum*. The schematic depicts the CRISPR/CAS locus of *C. violaceum*. Two CRISPR loci flank the six Cas genes (blue bars). The enlarged diagram shows the clamp formed in repetitive regions containing 28 nucleotides (brown bars) separated by a spacer sequence (green lozenge).

Our analysis identified the expression of two proteins from the Cas family (CRISPR-associated), exclusively in the treatment condition; namely, Csy4 and Csy1, encoded by the ORFs CV1751 and CV1754, respectively (Supplementary Table S4). The CRISPR (clustered, regularly interspaced or short palindromic repeats) is a mechanism providing resistance against invasive nucleic acids such as phages, conjugative plasmid and transposable elements. This bacterial “immune system” consists of a CRISPR RNA (crRNA) processing mechanism by endoribonuclease from the Csy family, generating the CRISPR^66^. Two distinct phases have been identified to this defense mechanism: (i) adaptation to initial contact with the foreign genetic element, where the Cas protein recognizes the foreign nucleic acid, followed by its incorporation into the 5′ ends of the CRISPR loci; and (ii) interference on re-exposure to the foreign nucleic acid, where a complex is formed consisting of ribonucleoprotein associated to short guide RNAs derived from CRISPRs and other proteins that are involved in destroying this material. The presence of Cas proteins in the proteome of *C. violaceum* indicates the existence of the CRISPRs/Cas system in this organism. In *C. violaceum*, this mechanism displays similar genome organization to that found in *Pectobacterium atrosepticum*^67^ and *Pseudomonas aeruginosa*^68^ (Fig. 4). The genome contains 25 repetitive regions containing 28 nucleotides and interspersed by spacer regions (consisting of viral genetic material), as well as an operon composed of six ORFs (CV_1749 to CV_1754) that encode proteins from the Cas family (Fig. 4). Activation of this mechanism under stress seems to be essential to the survival of this microorganism, given that oxidative stress activates the lytic pathway of several prophages^63^.

#### Differentially abundant proteins

TFold analysis pinpointed 14 differentially abundant proteins (Table 2). Supplementary Fig. S5 is a graphic representation of the TFold analysis, which maps proteins in accordance with the p-value for differential expression and its change in number of times. Of the 14 proteins, only three were down-regulated and 11 exhibited a statistically significant increase in expression after treatment. Proteins with increased expression were five dehydrogenases (GlpD, AldB, Ald, CV_2022 and CV_2037, and BetA), four prophage proteins (CV_0425, CV_0409, CV_0350 and CV_2128), one hypothetical (CV_0837) and one with other functions (MetY). Dehydrogenases are oxidoreductase enzymes that catalyze the oxireduction of a substrate by a reduction reaction, transferring one or more hydrides (H^−^) to an acceptor molecule as NAD^+^, NADP or FAD^69^. Increased expression of five dehydrogenases shows that *C. violaceum* requires an increased energy demand in the intracellular environment to combat stress caused by hydrogen peroxide. The detected dehydrogenases produce intermediate in the glycolytic pathway and the citric acid cycle, and also produces NADH^+^ that will feed the oxidative phosphorylation increasing the production of ATP.

The ORF CV_3027, which is annotated as a likely ferredoxin, decreased its abundance in 3.7 fold, being the most reduced expression (Table 2). In the *C. violaceum* genome, it appears to form part of the operon consisting of 4 ORFs (CV_3027 to CV_3030), all annotated and with functions related to iron homeostasis, except for CV_3029, whose function remains unknown. Expression of other members of this operon also varies. CV_3028 emerges only under control conditions. CV_3029 showed a statistically non-significant decrease and CV_3030 was not detected in this analysis. In bacteria, the hydrogen peroxide response is mediated by modifications in iron homeostasis^18^. Stress provoked by peroxide leads to changes in the distribution of this metal in cytosol in order to reduce damage caused by free iron ions. The intracellular decline in iron levels occurs via repression of metal uptake, as well as storage and sequestration action of proteins and incorporation into metalloproteins^18^. Metalloproteins, such as the ones that are part of energetic metabolism, were detected in the H_2_O_2_ responsive proteome although with no significant increase in their expression. However, the low increase in their expression could ameliorates damage caused by ROS by incorporating iron on their catalytic core, as suggested by Lima and colleagues^15^.

## Concluding Remarks

As far as we know, there are four proteomic studies of *C. violaceum* submitted to different kind of stresses^12^,^13^,^14^,^15^. Our analysis brings, by far, the most representative proteome of this bacterium, comprising a coverage of more than 30% of predicted ORFs, while the other studies, taken together, represents less than 8%. This proteome representation contributes to a better understanding of general environmental stress acclimation and may be used in further proteome studies for comparison purpose. Beside this, we have shown the evidence, in protein level, of many hypothetical ORFs that may have their role investigated in further analysis.

## Methods

### Bacterial strain, maintenance and growth conditions

The bacterial strain used in this study was *Chromobacterium violaceum* ATCC 12472. It was inoculated into Petri dishes with LB (Lysogeny-Broth) agar and incubated at 28 °C for 24 h. Individual colonies were aerobically pre-inoculated into 50 mL Falcon tubes containing 5 mL aliquots of liquid LB and submitted to agitation at 200 rpm and 28 °C for 16 hours. Pre-inoculated samples were transferred to 250 mL Erlenmeyer flasks in a fresh medium at a ratio of 10:1 (final volume of 50 mL culture). Bacteria were cultivated under the same conditions until reaching the exponential growth phase in an optical density (OD) at 600 nm of 0.4 – 0.5, which is approximately in 2 hours. Control samples remained under growth conditions and for treatment these were added to the 8 mM H_2_O_2_ cultures, continuing cultivation for two hours. Bacteria were cultivated and treated in three independent experiments.

In order to determine the effect of hydrogen peroxide on the growth rate of *C. violaceum*, bacteria were cultivated in liquid LB with or without 8 mM of hydrogen peroxide, at 200 rpm and 28 °C. Growth was monitored by measuring the OD_600_ of cultures at 15, 30, 60, 120, and 240 minutes.

Growth curve by CFU (colony-forming unity) count was performed. Treatment and growth conditions were as described above. Samples were collected at 0, 1, 2 and 4 hours of growth, with plating dilutions varying from 10^−5^ to 10^−7^. 100 μL of each sample was plated and the bacteria grew for 24 h prior colony counting using Scan 1200 from Intersicence. Finally, data was normalized to number of colonies in 10^−6^ dilution. Three biological replicates and two technical replicates were used.

### Protein extraction

Fifty mL of cell culture were centrifuged at 2,880 × g and 4 °C for 10 min. The precipitate was then re-suspended in 700 μL of solubilizing buffer (30 mM of Tris-HCl pH 8.0, 0.05% CHAPS, 7 M of urea, 1 M of thiourea, 50 mM of DTT) and stored at −20 °C. Total proteins were quantified according to Bradford^70^.

### Analysis of catalase activity levels

Catalase activity levels were determined with commercial Catalase Assay Kits (Catalogue no. 707002-Cayman Chemical Company, USA) according to the manufacturer’s instructions. This assay is based on the reaction of catalase with methanol in the presence of hydrogen peroxide. The formaldehyde produced is measured upon its reaction with Purpald chromogen which specifically forms a bicyclic compound that changes to colorless to purple color upon oxidation. Catalase activity was measured using total proteins extract as described above and was calculated in nmol.min/μg of protein. To normalize the enzyme activity, the concentration of total protein extract was used. The experiment was performed in three biological replicates.

### Polyacrylamide gel electrophoresis (SDS-PAGE)

A total of 20 μg of protein was submitted to SDS-PAGE 12% in a Mini-Protean electrophoresis module (Bio-Rad). Precision Plus Proteins WesternC Standard (Bio-Rad) was used as marker. The gel was further stained with Coomassie Colloidal G-250 (Sigma-Aldrich).

### In-gel Digestion

In-gel digestion was made based on Shevchenko and colleagues^71^. Each gel lane was cut into 9 slices, which were then trimmed into approximately 1 mm^3^ pieces (Supplementary Fig. S6). For destaining, the gel was washed 3 × 30 minutes with 100 μL of 50% acetonitrile (ACN) solution and 10 mM of NH_4_HCO_3_, pH 8.0 and then dehydrated with 100 μL of ACN for 10 min. Disulfide bridges were reduced using 100 μL of 10 mM dithiothreitol (DTT) in 100 mM NH_4_HCO_3_ for 30 min at room temperature followed by dehydration for 10 min with 100 μL of 100% acetonitrile and alkylation with 50 μL of idoacetamide (IAM) 55 mM/10 mM NH_4_HCO_3_ for 30 min in the dark. The gel was dehydrated under the same conditions and dried with *Concentrator Plus* (Eppendorf).

15 μL of 13 ng μL^−1^ trypsin solution (Trypsin Promega Sequencing Grade Modified) in 10% NH_4_HCO_3_ 100 mM and 10% acetonitrile was added to the dried gels in an ice bath for 30 minutes and then incubated at 37 °C for 16 hours.

Tryptic peptides were extracted from the gel by adding 30 μL of extraction solution (95% ACN and 5% formic acid at 0.1%) and placed in a shaker under low agitation for 15 minutes at room temperature. Next, the entire solution was removed and transferred to new tubes and dried with *Concentrator Plus* (Eppendorf). Prior to being placed in the mass spectrometer, the samples were reconstituted in 10 μL of 0.1% formic acid and sonicated for 10 min.

### LC–MS/MS and data analysis

We used a reversed phase EASY-nano LC system (Proxeon, Odense, Denmark) coupled to an LTQ-Orbitrap *Velos* mass spectrometer (Thermo Fisher Scientific, San Jose, CA). Each hydrolysate was loaded in two replicates (technical replicates) into an 18 cm fused-silica emitter, 100 μm I.D., packed in-house with ReproSil-Pur C18-AQ reversed phase 3 μm resin (Dr. Maisch GmbH, Germany). Peptides were eluted using a gradient from 100% phase A (5% acetonitrile +0.1% formic acid) to 50% phase B (80% acetonitrile +0.1% formic acid) in 70 minutes; 50% to 60% phase B in 20 minutes; 60% phase B to 100% in 15 minutes and 100% phase B in a further 15 minutes at a flow rate of 200 nL/min. Ion mass spectra were obtained in positive mode by automated MS scanning and tandem MS/MS. Each MS scan collected in the Orbitrap at m/z 300–2000 and resolution of 60,000, was followed by MS/MS of the ten most intense ions in the LTQ. Fragmentation in the LTQ was performed by collision-induced dissociation (CID) with a dynamic exclusion of 60 seconds and normalized collision energy of 35.

ProLuCID v1.3 search engine was used to perform peptide spectrum matching against the *Chromobacterium violaceum* proteins database downloaded from [http://www.ncbi.nlm.nih.gov/protein, September 2011]^72^. Search parameters considered fully tryptic peptides with up to two missed cleavages, oxidation of methionine as variable and carbamidomethylation as fixed modifications, and a peptide tolerance of 50 ppm. The validity of the Peptide Sequence Matches (PSM) was assessed using the Search Engine Processor v. 2.2.0.2 (SEPro)^73^. Identifications were grouped by charge state (+2 and ≥ +3), resulting in four distinct subgroups. For each result, the ProLuCID XCorr, DeltaCN, and ZScore values were used to generate a Bayesian discriminating function. A cutoff score was established to accept a false-discovery rate (FDR) of 1% based on the number of decoys. This procedure was independently performed on each data subset, resulting in a false-positive rate that was independent of charge state. A minimum sequence length of 6 amino acid residues was required. Results were post-processed to accept PSM with less than 8 ppm. Proteins were grouped according to the maximum parsimony by the bipartite graph approach^74^.

### Defining differential abundance of proteins using TFold

The PatternLab TFold module^75^,^76^ was used to investigate differentially expressed proteins under control conditions and treated with 8 mM of H_2_O_2_. Different from other algorithms, TFold uses a variable fold-change cutoff that takes into account a theoretical FDR estimator that maximizes the number of identifications, detecting low abundant proteins. Only proteins present in at least two biological replicates were considered for this analysis.

### Functional annotation and sequence analysis

The proteins detected, characterized as hypothetical and/or conserved hypothetical proteins were re-annotated using BLASTp (www.ncbi.nlm.nih.gov) for function assignment. Criteria defined for this analysis were *E* value 10^–15^, 30% identity and 60% coverage. For some proteins we also performed multiple alignment using the ClustralW program (http://www.ebi.ac.uk/Tools/msa/clustalw2/). If the hypothetical protein aligned with a known protein with the up-mentioned criteria, we re-annotated it. The Functional Annotation Tool was used to classify proteins into functional categories, available through the DAVID Bioinformatics Database (http://david.abcc.ncifcrf.gov)^34^.

### RNA extraction of *C. violaceum* and synthesis of cDNA

Isolated colonies of *C. violaceum* were previously inoculated and the treatment in the presence and absence of 8 mM H_2_O_2_ was made during two hours as mentioned above. Later, total RNA was extracted using RNAspin Mini Isolation Kit, according to manufacture’s instructions (GE, catalog number 25-0500-72).

The synthesis of cDNA was further made using the up-mentioned RNA. High capacity cDNA reverse transcriptase kit was used according to manufacturer’s instructions (Applied Biosystems, 4368814).

### Validation of protein levels with real-time PCR

To validate the protein levels of our analysis we chose the bioC that was exclusively identified in the treatment condition and ferredoxin (downregulated upon H_2_O_2_ exposure) genes to be analyzed with real-time quantitative PCR. To this, ten nanograms of cDNA (produced as mentioned above) and a final concentration of 0, 25 nM of each primer were applied in a 10-μl reaction using Power SYBR Green PCR Master Mix (Applied Biosystems, 4367659) in an One-Step cycling with the following conditions: 95 °C for 3 min, 40 cycles of denaturation at 95 °C for 3 sec, annealing/extension at 60 °C for 15 sec. To normalize the expression, 16S rRNA gene was used as endogenous control. ΔΔCt was used to assess the relative quantification of the mentioned genes. Primer 3 (v. 0.4.0) tool was used to design the primers (Supplementary Table S6). Statistical analysis was performed according to the t-test. Results were considered significant for *p* < 0.05.

## Additional Information

**How to cite this article**: Lima, D. C. *et al*. GeLC-MS-based proteomics of *Chromobacterium violaceum*: comparison of proteome changes elicited by hydrogen peroxide. *Sci. Rep.* **6**, 28174; doi: 10.1038/srep28174 (2016).

## Supplementary Material

Supplementary Information

Supplementary Table S1

## Figures and Tables

**Table 1:** List of the 30 Proteins with most spectral counts in the proteome of *C. violaceum*.

UniProt accession	Spectral Counts in Control	Spectral Counts in Treatment	Description	Functional categories
Q7NSK0	622	730	Porin signal peptide protein GN = *ompC*	Porin, Gram-negative type
Q7NQE6	605	646	DNA-directed RNA polymerase subunit beta GN = *rpoB*	Purine metabolism
Q7NY63	434	650	Formate C-acetyltransferase GN = *pflB*	Pyruvate metabolism
Q7NXI3	513	513	Chaperone protein DnaK GN = *dnaK*	Protein folding
Q7NQE7	513	501	DNA-directed RNA polymerase subunit beta’ GN = *rpoC*	Purine metabolism
Q7NQX1	463	549	60 kDa chaperonin 2 GN = *groL2*	Protein folding
Q7NTF3	585	424	Probable porin signal peptide protein GN = CV_3104	Porin, Gram-negative type
Q7NTK8	525	469	30S ribosomal protein S1 GN = *rpsA*	Ribosome
Q7M7F1	443	489	Elongation factor Tu GN = *tuf1*	Translation
Q7NQF0	399	372	Elongation factor G GN = *fusA*	Translation
Q7NTH1	368	403	Glutamate dehydrogenase GN = *gdhA*	Alanine, aspartate and glutamate metabolism
Q7NZ25	225	474	Probable binding protein component of ABC dipeptide transporter GN = CV_1097	ABC transporters
Q7NQH6	266	385	30S ribosomal protein S4 GN = *rpsD*	Ribosome
Q7P095	254	368	ATP synthase subunit beta GN = *atpD*	Oxidative phosphorylation
Q7NZE2	300	311	Probable phosphoenolpyruvate-protein phosphotransferase GN = CV_0980	Carbohydrate transport and metabolism,
Q7NX40	238	327	Protein kinase GN = *prkA*	Signal transduction mechanisms
Q7NY13	238	312	Translation initiation factor IF-2 GN = *infB*	Translation
Q7P0P0	273	254	Pyruvate dehydrogenase E1 component GN = *aceE*	Glycolysis/Gluconeogenesis
Q7NVZ3	223	285	Elongation factor Ts GN = *tsf*	Translation
Q7NV74	183	269	Aconitate hydratase 2 GN = *acnB*	Glyoxylate and dicarboxylate metabolism
Q7NTP3	186	258	Flagellin D GN = *flaD*	Flagellar assembly
Q7NWN7	206	224	Chaperone protein ClpB GN = *clpB*	Nucleotide binding
Q7NQM5	133	286	Aspartate ammonia-lyase GN = *aspA*	Tricarboxylic acid cycle
Q7NQE3	217	194	50S ribosomal protein L1 GN = *rplA*	Ribosome
Q7NYH1	166	245	Inosine-5′-monophosphate dehydrogenase GN = *guaB*	Purine metabolism
Q7NRP1	172	238	Probable peroxidase GN = CV_3739	Oxidative metabolism
Q7NUY8	191	213	Trigger factor GN = *tig*	Protein folding
Q7NYY5	211	186	Acetaldehyde dehydrogenase GN = *adhE*	Glycolysis/Gluconeogenesis
Q7NX09	196	194	Probable ribonuclease E GN = CV_1820	RNA degradation,
Q7NY10	183	204	Polyribonucleotide nucleotidyltransferase GN = *pnp*	Purine metabolism

The proteins are represented in descending order. GN = Gene name.

**Table 2:** Differentially abundant proteins selected by the TFold Analysis.

Uniprot accession	Gene	Fold change	Description
Q7P105	CV_0409	6, 3	Probable bacteriophage tail sheath protein
Q7NYH4	*bet*A	5, 0	Glucose-methanol-choline oxidoreductase
Q7P164	CV_0350	4, 4	Probable phage sheath protein
Q7NW62	CV_2128	4, 3	Probable bacteriophage tail sheath protein
Q7P1F9	*glp*D	4, 1	Glycerol-3-phosphate dehydrogenase
Q7P0Z0	CV_0425	4, 0	Putative uncharacterized protein
Q7NWP7	*met*Y	3, 7	O-acetylhomoserine (Thiol)-lyase
Q7NZT4	CV_0837	3, 6	Putative uncharacterized protein
Q7NWF3	CV_2037	3, 2	Probable dihydrolipoamide dehydrogenase
Q7P121	*ald*B	2, 9	Aldehyde dehydrogenase (NAD)
Q7NWG7	*ald*	2, 6	Alanine dehydrogenase
Q7P1P4	*fad*H	−3, 1	2,4-dienoyl-CoA reductase FadH1
Q7NYM4	CV_1250	−3, 4	UPF0246 protein
Q7NTM7	CV_3027	−3, 7	Probable ferredoxin

These proteins presented the most significant changes in their abundance (p < 0.01). A positive fold change indicates a higher abundancy in the treatment condition.
