# Supplementary material for: GeLC-MS-based proteomics of Chromobacterium violaceum: comparison of proteome changes elicited by hydrogen peroxide
Source: Sci Rep. 2016 Jun 20;6:28174. doi: 10.1038/srep28174 (PMC4913304; doi:10.1038/srep28174)
Supplement: Supplementary Information [file srep28174-s1.pdf]

Supplementary Information

GeLC-MS-based proteomics of *Chromobacterium violaceum*: comparison of proteome changes elicited by hydrogen peroxide

Lima, D.C[1], Duarte, F.T[2], Medeiros, V.K.S[3], Carvalho, P.C[4]., Nogueira, F.C.S[5], Araujo G.D.T[5], Domont G.B[5], Batistuzzo de Medeiros, S.R[1]

1. Laboratório de Biologia Molecular e Genômica, Centro de Biociências, Universidade Federal do Rio Grande do Norte, Campus Universitário s/n, Lagoa Nova, Natal, RN, 59072-970 Brazil
2. Instituto Federal de Educação, Ciência e Tecnologia do Rio Grande do Norte (IFRN) - RN, Brazil
3. Colegiado de Medicina, Universidade Federal do Vale do São Francisco, Avenida José de Sá Maniçoba, S/N, Centro, Petrolina, PE, 56304-917, Brazil
4. Laboratório de Proteômica e Engenharia de Proteínas, Instituto Carlos Chagas, Fiocruz, Avenida Prof. Algacyr Munhoz Mader, Curitiba, PR, 8135010, PR, Brazil
5. Unidade Proteômica, Laboratório de Química de Proteínas, Bloco A - sala 543, Centro de Tecnologia, Universidade Federal do Rio de Janeiro, Av. Athos da Silveira Ramos, 149, Cidade Universitária, Rio de Janeiro, RJ, 21941 - 909- Brazil.

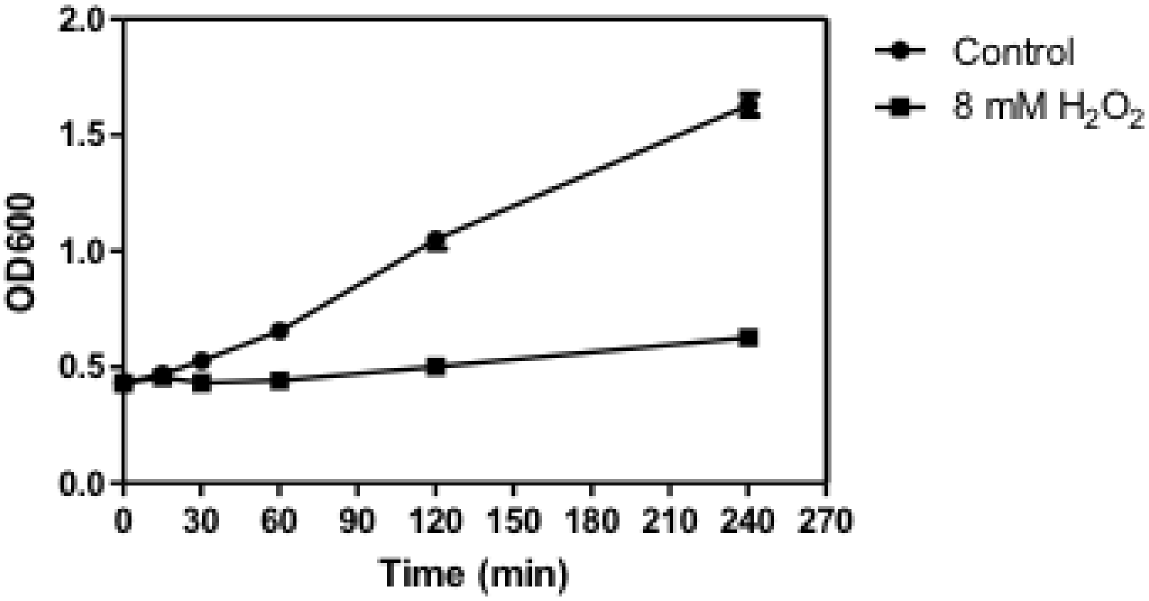

Supplementary Figure S1: Cellular growth curve. Effect of treatment with H_2O_2 on the cellular growth curve of *C. violaceum*. Time “0” is the OD between 0.4-0.5 when the hydrogen peroxide treatment has begun.

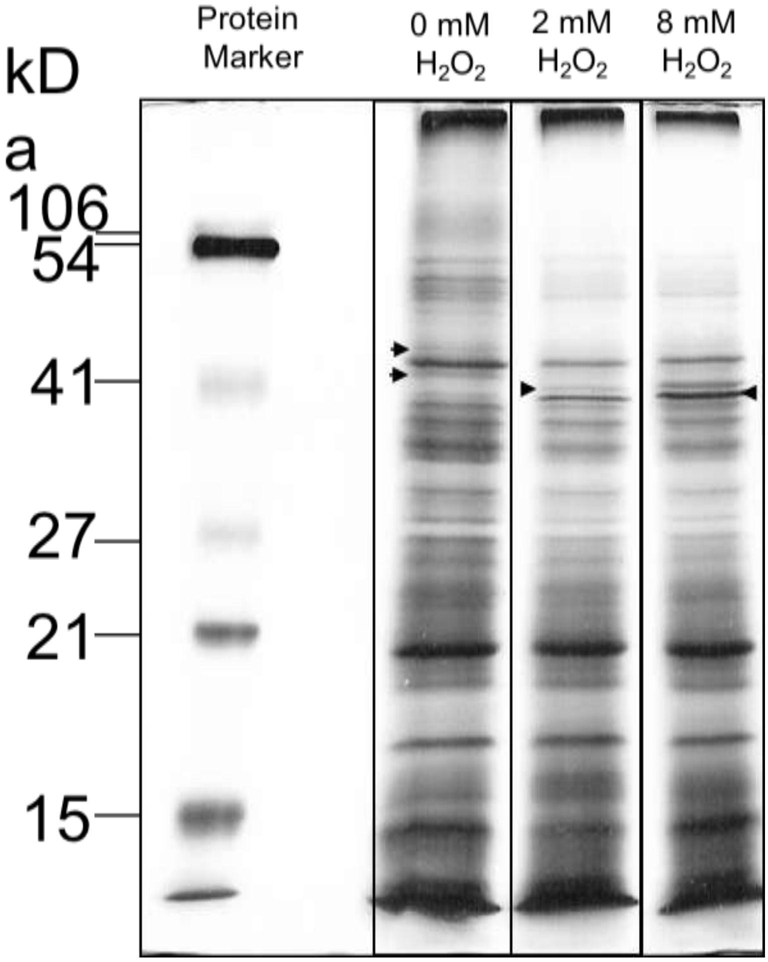

Supplementary Figure S2: SDS-PAGE with total proteins from *C. violaceum* extracted in the absence (lane 2) and in the presence of 2 mM (lane 3) and 8 mM (lane 4) of hydrogen peroxide. The black arrows indicate protein bands that changed their patter after oxidative stress exposure.

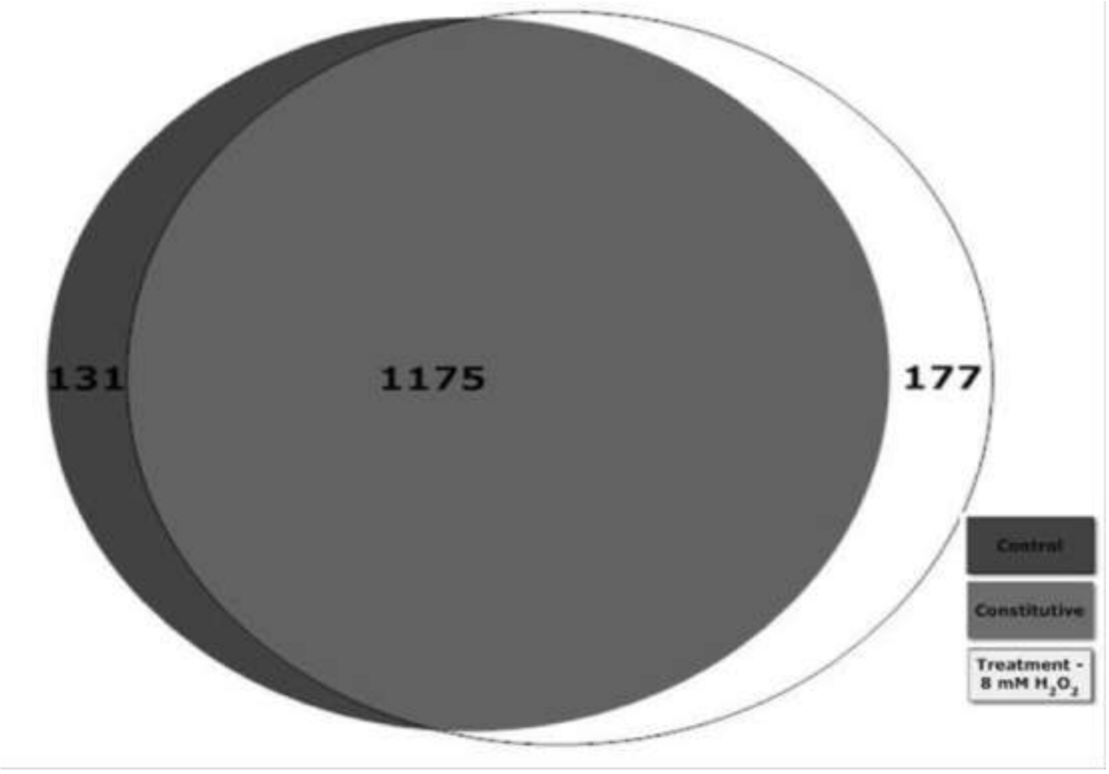

Supplementary Figure S3: Venn diagram of proteins identified in the three repetitions of treatments with 8 mM of H_2O_2 and controls in the absence of H_2O_2. Dark grey: exclusively control proteins; White: specific proteins of cells treated with H_2O_2; Light grey: proteins abundance under both conditions. Only proteins detected in the three biological replicates of a specific condition were considered.

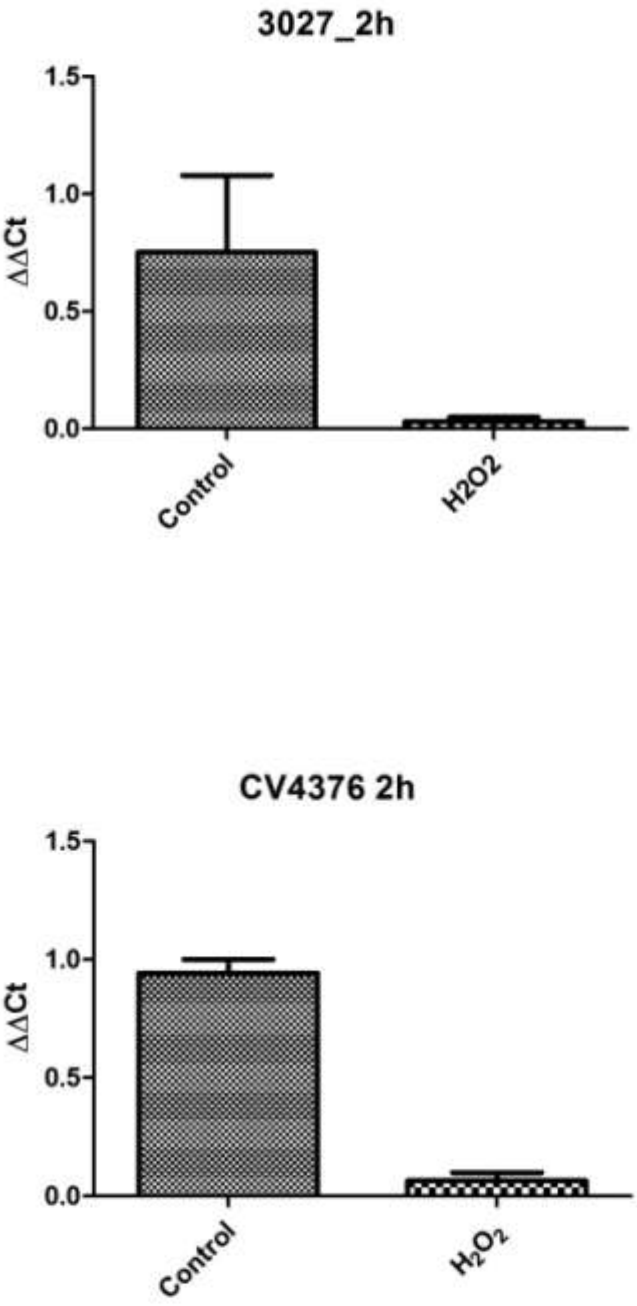

Supplementary Figure S4 : Real-time PCR of ferredoxin (CV_3027) and *bioC* (CV_4376) after 2 hour exposure with 8 mM H_2O_2.

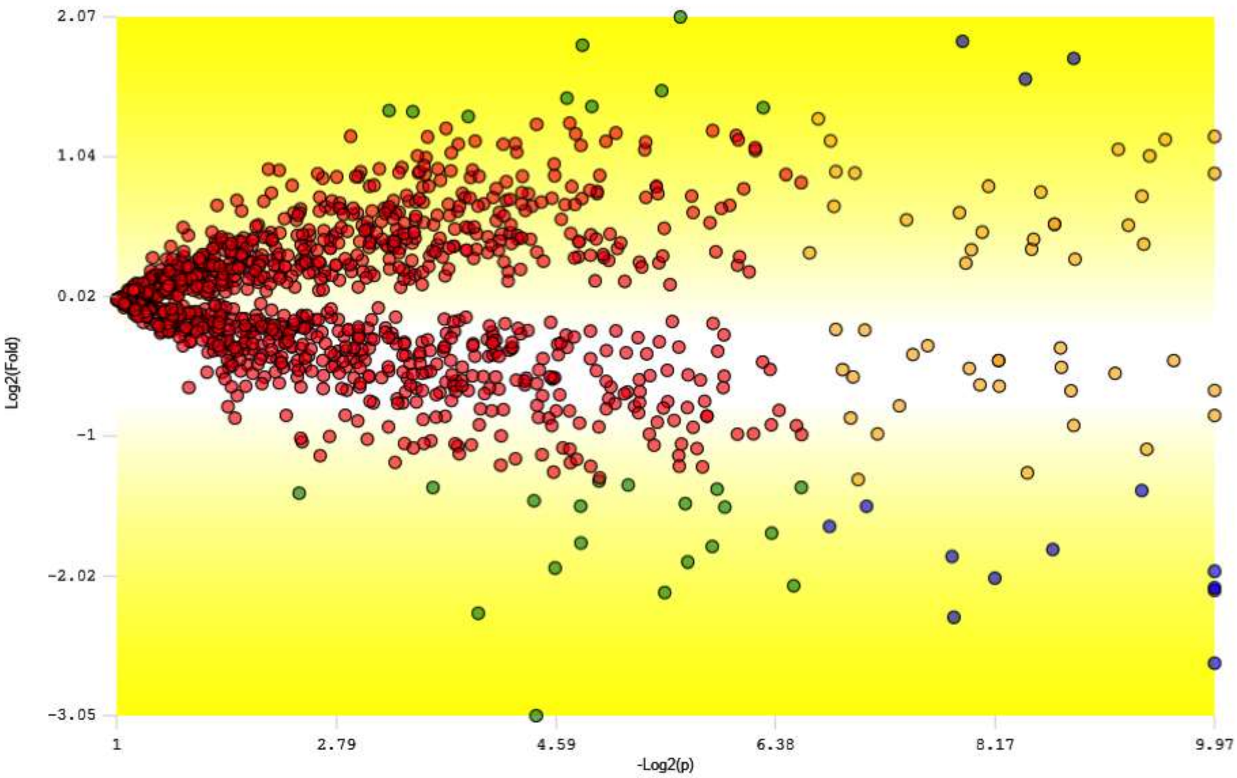

Supplementary Figure S5: Differential expression of *C. violaceum* proteins. Graph shows TFold values and compares protein level under treated and oxidative stress conditions induced by 8 mM H_2O_2. This analysis considers only proteins detected in at least two biological replicates. Each dot symbolizes a protein; ordinates represent protein abundance change and abscissas the p-value of the t-test. The fold change value is the number of times obtained by calculating the ratio of average relative peptide quantification values for each condition studied. Parameters established for this analysis were q<0.01 and variable fold-change cutoff, with blue dots identifying proteins that satisfy these two parameters. Proteins represented by orange dots were identified as statistically different, although they do not comply with variable cutoff parameters for changes in number of times. Those depicted by orange dots satisfy the variation cutoff for number of times, but do not meet statistical criteria. Red dots do not comply with either of the criteria.

The theoretical BH false-positive estimator (q <0.05) indicates that proteins identified as differentially expressed are in fact so. There are 1000 dots in the graph: 14 blue, 28 green, 51 orange and the remainder red.

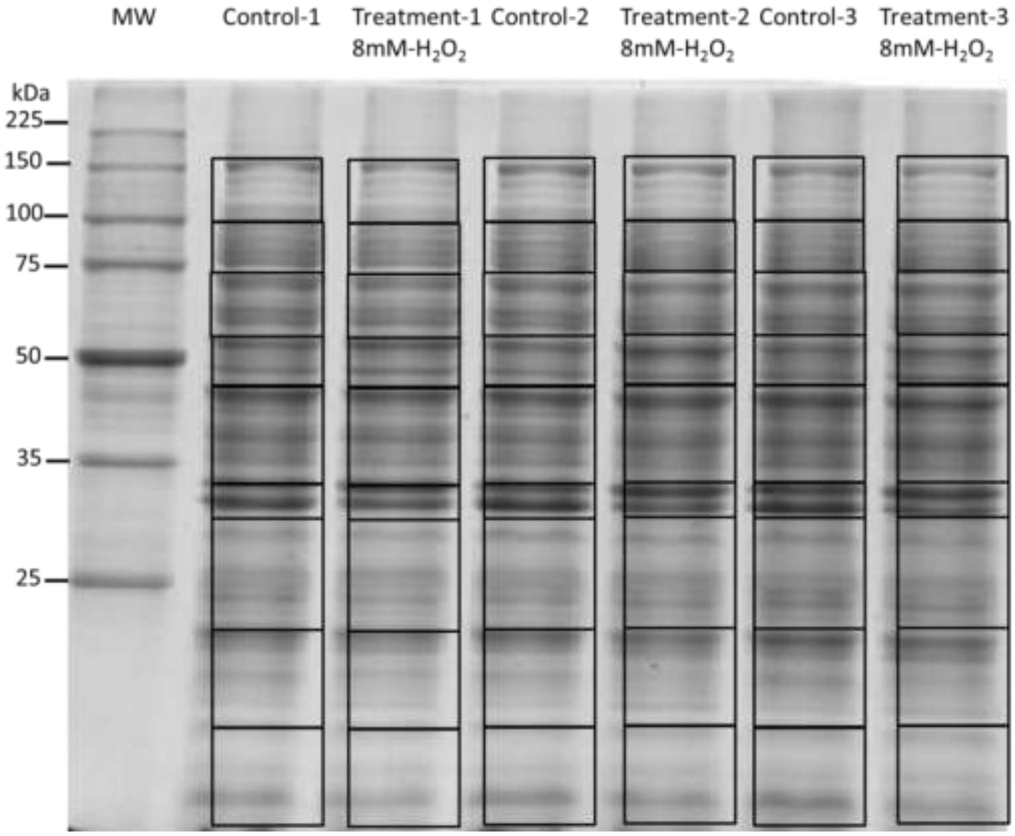

Supplementary Figure S6: SDS-PAGE of total proteins extracted from *C. violaceum* in the absence and presence of 8 mM H_2O_2. Three biological replicates for each condition were used. Prior to trypsin digestion, each lane was sliced in 9 pieces (squares in the figure).

Supplementary Table S1*: Proteins, with their respective signals, detected in both treatment and control conditions.

* This table is in a separated .xlf file.

Supplementary Table S2: Re-annotation and genomic context of hypothetical and/or conserved hypothetical proteins detected exclusively in oxidative stress condition.

ORF	Protein	Genome context (operon)	E-value	Organism	Protein	Query coverage (%)	Identity (%)	OBS
**CV_0424**	Putative uncharacterized protein	CV_0421 CV_0422 CV_0423 CV_0424 CV_0425 CV_0426	$8e^{-44}$	*Pseudomonas putida* GB-1	putative pyocin R2_PP, tail fiber protein	96	40	Probable viral protein
**CV_0425**	Putative uncharacterized protein	CV_0421 CV_0422 CV_0423 CV_0424 CV_0425 CV_0426	-	-	hypothetical protein	-	-	Probable viral protein

**CV_3909**	conserved hypothetical protein	CV_3909<br>CV_3910<br>CV_3911	-	-	hypothetical protein			
**CV_0338**	conserved hypothetical protein	CV_0338<br>CV_0339			hypothetical protein			
**CV_3452**	conserved hypothetical protein	CV_3450<br>CV_3451<br>CV_3452<br>CV_3453<br>CV_3454	$5e^{-73}$	*Pseudogulbenkiania* sp. NH8B	peptidase M22 glycoprotease	100	57	Probable viral protein
**CV_0256**	conserved hypothetical protein	-	$6e^{-25}$	*Lactococcus lactis* subsp. lactis KF147	tRNA-binding protein	93	33	
**CV_3265**	hypothetical protein	-	-	-	hypothetical protein	-	-	-
**CV_1754**	conserved hypothetical protein	CV1749<br>CV1750<br>CV1751<br>CV1752<br>CV1753<br>CV1754<br>CV1755<br>CV1756	0.0	*Laribacter hongkongensis* HLHK9	CRISPR-associated protein, Csy1 family	92	66	
**CV_1751**	conserved hypothetical protein	CV1749<br>CV1750	$3e^{-50}$	*Verminephrobacter aporrectodeae* subsp. tuberculatae At4	CRISPR-associated Csy4 family protein	97	49	

		CV1751<br>CV1752<br>CV1753<br>CV1754<br>CV1755<br>CV1756						
**CV_4246**	conserved hypothetical protein	CV_4246<br>CV_4247	-	-	conserved hypothetical protein	-	-	-
**CV_3475**	hypothetical protein	-	-	-	hypothetical protein	-	-	
**CV_3151**	hypothetical protein	-	-	-	hypothetical protein	-	-	
**CV_0714**	conserved hypothetical protein	-	$1e^{-84}$	*Rhodanobacter* sp. 2APBS1	NmrA family protein	100	57	
**CV_3236**	conserved hypothetical protein	-	$3e^{-99}$	*Acinetobacter baumannii* ATCC 19606	ThiJ/PfpI domain-containing protein	69	57	
**CV_0348**	conserved hypothetical protein	CV_0345<br>CV_0346<br>CV_0347<br>CV_0348	$9e^{-40}$	*Burkholderia* sp. JV3	peptidoglycan-binding domain 1 protein	31	42	Probable viral protein
**CV_2925**	conserved hypothetical protein	CV_2926<br>CV_2927	$1e^{-165}$	*Hylemonella gracilis* ATCC 19624	exopolyphosphatase-like protein	99	72	-
**CV_0342**	Conserved hypothetical protein	CV_0342<br>CV_0343<br>CV_0344	$5e^{-77}$	*Laribacter hongkongensis* HLHK9	phage tail protein	96	66	Probable viral protein
**CV_1311**	hypothetical protein	_	-	-	hypothetical protein	-	-	-

**CV_3160**	conserved hypothetical protein	-	$3e^{-48}$	*Achromobacter xylosoxidans* AXX-A	MarR family transcriptional regulator	91	54	
**CV_1144**	conserved hypothetical protein	-	$5e^{-54}$	*Delftia* sp. Cs1-4	DinB family protein	94	49	
**CV_4296**	conserved hypothetical protein	-	-	-	conserved hypothetical	-	-	
**CV_0785**	conserved hypothetical protein	CV_0785 CV_0786	$4e^{-111}$	*Pseudogulbenkiania* sp. NH8B	methionine biosynthesis protein MetW	96	79	
**CV_3599**	hypothetical protein	CV_3599 CV_3560	-	-	hypothetical protein	-	-	Probable viral protein
**CV_0011**	hypothetical protein	CV_0011 CV_0012 CV_0013	-	-	hypothetical protein	-	-	
**CV_0212**	conserved hypothetical protein	-	$2e^{-42}$	*Yersinia enterocolitica* subsp. enterocolitica 8081	esterase YqiA	94	40	
**CV_3543**	conserved hypothetical protein	-	$8e^{-51}$	*Pseudogulbenkiania* sp. NH8B	sporulation domain containing protein	88	46	
**CV_2113**	hypothetical protein	CV_2112 CV_2113 CV_2114			hypothetical protein			
**CV_3581**	conserved hypothetical protein	-	0.0	*Lutiella nitroferrum* 2002	Cupin 4 family protein	99	68	Probable viral protein
**CV_0746**	hypothetical protein	-	-	-	hypothetical protein	-	-	

**CV_2902**	conserved hypothetical protein	-	-	-	conserved hypothetical protein	-	-	
**CV_3388**	conserved hypothetical protein	-	1e^{-32}	*Vibrio splendidus* ATCC 33789	hypothetical acetyltransferase	95	38	
**CV_3387**	conserved hypothetical protein	-	7e^{-118}	*Lutiella nitroferrum* 2002	segregation and condensation protein B	97	83	
**CV_2971**	hypothetical protein	-	-	-	hypothetical protein	-	-	
**Cv_2738**	conserved hypothetical protein	CV_2738 CV_2739 CV_2740	2e^{-178}	*Ralstonia solanacearum* CMR15	putative ATPase family associated with various cellular activities (AAA) protein	98	69	
**CV_0178**	conserved hypothetical protein	-	6e^{-107}	*Pseudogulbenkiania* sp. NH8B	alanine racemase domain containing protein	99	76	
**CV_2075**	conserved hypothetical protein	CV_2073 CV_2074 CV_2075	3e^{-138}	*Pseudogulbenkiania* sp. NH8B	molybdopterin binding domain containing protein	98	79	
**CV_0407**	hypothetical protein	CV_0407 CV_0408 CV_0409 CV_0410	3e^{-21}	*Ralstonia solanacearum* CFBP2957	phage protein	99	39	Probable viral protein
**CV_3155**	conserved hypothetical protein	-	1e^{-77}	*Burkholderia* sp. CCGE1002	diamine N-acetyltransferase	96		
**CV_0791**	conserved hypothetical protein	-	4e^{-94}	*Lutiella nitroferrum* 2002	ybaK/ebsC protein	98	84	
**CV_3157**	conserved hypothetical protein	CV_3157	3e^{-88}	*Pseudomonas brassicacearum* subsp.	kinase inhibitor	98	70	

		CV_3158		brassicacearum NFM421			
**CV_3425**	conserved hypothetical protein	-	$1e^{-22}$	*Mycobacterium kansasii* ATCC 12478	putative transcriptional regulatory protein	83	35
**CV_3201**	conserved hypothetical protein	CV_3201<br>CV_3202	$7e^{-59}$	*Ktedonobacter racemifer* DSM 44963	thioesterase superfamily protein	92	54
**CV_0330**	conserved hypothetical protein	-	-	-	conserved hypothetical protein	-	-
**CV_0565**	hypothetical protein	-	$7e^{-59}$	*Lutiella nitroferrum* 2002	Hemerythrin HHE cation binding domain protein	98	53
**CV_0156**	conserved hypothetical protein	-	$2e^{-91}$	*Lutiella nitroferrum* 2002	adenylate cyclase	100	82
**CV_3623**	conserved hypothetical protein	CV_3622<br>CV_3623	-	-	hypothetical protein	-	-
**CV_0053**	conserved hypothetical protein	CV_0050<br>CV_0051<br>CV_0052<br>CV_0053<br>CV_0054	-	-	- conserved hypothetical protein	-	-

Supplementary Table S3: Re-annotation and genomic context of hypothetical and/or conserved hypothetical proteins expressed exclusively in control condition.

ORF	Protein	Operon	E-value	Organism	Protein	Query coverage (%)	Identity (%)
**CV_1445**	hypothetical protein	-	-	-	hypothetical protein	-	-
**CV_1007**	conserved hypothetical protein	-	$7e^{-171}$	*Pseudogulbenkiania sp. NH8B]*	serine/threonine protein kinase	97	75
**CV_0635**	hypothetical protein	-	$6e^{-19}$	*Roseiflexus sp. RS-1*	putative PAS/PAC sensor protein	63	31
**CV_1727**	conserved hypothetical protein	-	$5e^{-70}$	*Paenibacillus sp. HGF7*	Cof-like hydrolase	96	44
**CV_3008**	hypothetical protein	-	$3e^{-30}$	*Burkholderia phymatum STM81*	response regulator receiver protein	98	33
**CV_0153**	conserved hypothetical protein	CV_0153<br>CV_0153	$2e^{-127}$	*Pseudogulbenkiania sp. NH8B*	peptidase M48, Ste24p	98	67
**CV_4304**	conserved hypothetical protein	-	$2e^{-44}$	*Lutiella nitroferrum 2002*	YfaZ family protein	100	48
**CV_2363**	conserved hypothetical protein	-	$2e^{-72}$	*Pseudogulbenkiania sp. NH8B*	GAF domain-containing protein	91	70
**CV_3513**	conserved hypothetical protein	CV_3513<br>CV_3514	-	-	conserved hypothetical protein	-	-
**CV_1711**	conserved hypothetical protein	CV_1709<br>CV_1710<br>CV_1711	-	-	conserved hypothetical protein	-	-

**CV_2561**	hypothetical protein	-	-	-	hypothetical protein	-	-
**CV_3785**	conserved hypothetical protein	-	$1e^{-118}$	*Pseudogulbenkiania sp. NH8B*	NAD-dependent epimerase/dehydratase	63	94
**CV_1784**	hypothetical protein	CV_1783<br>CV_1784	-	-	hypothetical protein	-	-
**CV_0286**	conserved hypothetical protein	-	-	-	conserved hypothetical protein	-	-
**CV_2486**	conserved hypothetical protein	CV_2485	-	-	conserved hypothetical protein	-	-
**CV_4122**	hypothetical protein	CV_4122<br>CV_4123<br>CV_4124	$1e^{-52}$	*Acidovorax delafieldii 2AN*	acetyltransferase	92	51
**CV_3423**	conserved hypothetical protein	-	-	-	conserved hypothetical protein	-	-
**CV_2149**	hypothetical protein	-	-	-	hypothetical protein	-	-
**CV_1753**	conserved hypothetical protein	CV_1751<br>CV_1752<br>CV_1753<br>CV_1754	$3e^{-130}$	*Laribacter hongkongensis HLHK9*	CRISPR-associated protein, Csy2 family	96	59
**CV_2799**	conserved hypothetical protein	CV_2798<br>CV_2799	$3e^{-63}$	*Pseudomonas syringae pv. morsprunorum str. M302280PT*	non-ribosomal peptide synthetase SyfB	71	41
**CV_0913**	conserved hypothetical protein	-	-	-	conserved hypothetical protein	-	-
**CV_3937**	conserved hypothetical protein	CV_3936<br>CV_3937	$4e^{-123}$	*Methylovorus glucosetrophus SIP3-4*	Succinylglutamate desuccinylase/aspartoacylase	96	58

**CV_2329**	Putative uncharacterized protein	-	$2e^{-167}$	*Lutiella nitroferrum 2002*	dihydrouridine synthase DuS	98	75
**CV_3974**	conserved hypothetical protein	CV_3973<br>CV_3974<br>CV_3975	-	-	hypothetical protein	-	-
**CV_2915**	hypothetical protein	CV_2915<br>CV_2916<br>CV_2916	$3e^{-155}$	*Pseudogulbenkiania sp. NH8B*	permease, YjgP/YjgQ family	95	64
**CV_2059**	conserved hypothetical protein	CV_2058<br>CV_2059<br>CV_2060	$8e^{-60}$	*Lutiella nitroferrum 2002*	anti sigma-E protein, RseA	100	58
**CV_1999**	hypothetical protein	CV_1999<br>CV_2000	$2e^{-107}$	*Lutiella nitroferrum 2002*	20S proteasome A and B subunits	99	81
**CV_3720**	hypothetical protein	CV_3719<br>CV_3720	$1e^{-161}$	*Pseudogulbenkiania sp. NH8B*	hydrolase, TatD family	100	83
**CV_2003**	hypothetical protein	-	-	-	hypothetical protein	-	-
**CV_0373**	hypothetical protein	-	-	-	hypothetical protein	-	-
**CV_3724**	hypothetical protein	CV_3721<br>CV_3722<br>CV_3723<br>CV_3724	$6e^{-139}$	*Lutiella nitroferrum 2002*	aminodeoxychorismate lyase	91	64

Supplementary Table S4: Protein Functional Category Classification, uniquely detected in oxidative stress condition.

Pathway classification				
Carbohytrate metabolism	**Gene name**	**Description**	**Uniprot code**	**ORF**
**Amino sugar and nucleotide sugar metabolism**	*graD*	Probable Bifuncional: UDP-N-acetylglucosamineglucose-1-phosphate thymidylyltransferase; Glucosamine-1-phosphate	Q7MBE7	CV_3103
	ubiB	Probable ubiquinone biosynthesis protein UbiB	Q7NZD1	CV_3784
**Glyoxylate and dicarboxylate metabolism**				
	gph	probable phosphoglycolate phosphatase	Q7NX12	CV_1817
**Glycoside hydrolase**				
	CV_2894	Probable endoglucanase-related protein	Q7NU09	CV_2894
Metabolism Energy				
**General metabolism Energy**				
	adhC	Alcohol dehydrogenase class III	Q7P029	CV_0740
	CV_1452	Probable iron-sulfur 4Fe-4S ferredoxin transmembrane protein	Q7NY23	CV_1452
**Nitrogen Metabolism**				
	tap	probable methyl-accepting chemotaxis protein	Q7NWR8	CV_1912
	narH	Nitrate reductase	Q7NV04	CV_2542
**Oxidative phosphorylation**				
	petB	Cytochrome b	Q7NQX8	CV_4007
	CV_3278	Probable cytochrome b561	Q7NSZ1	CV_3278
**Oxidoreductase**				
	yedY	Sulfoxide reductase catalytic subunit yedY	Q7NZY0	CV_0789
	msrA	Peptide methionine sulfoxide reductase MsrA	Q7NVL7	CV_2325
	ptr1	Probable pteridine reductase	Q7NQK5	CV_4132
**dTDP-4-dehydrorhamnose reductase, NAD(P)-binding domain**				
	CV_0714	NmrA family protein	Q7P054	CV_0714
	CV_2738	putative ATPase family associated with various cellular activities	Q7NUG0	CV_2738
Metabolism of cofatctor end vitamins				
**Co-factors**				
	thiE	Thiamine-phosphate pyrophosphorylase	Q7P1R3	
	folE	GTP cyclohydrolase 1	Q7NYZ2	CV_1130
**Folate biosynthesis**				
	folC	Folylpolyglutamate synthase/dihydrofolate synthase	Q7NV28	CV_2518

Porphyrin and chlorophyll metabolismo				
	hemF	Coproporphyrinogen-III oxidase, aerobic	Q7P012	CV_0757
**Thiamine metabolism**				
	CV_3611	Probable bifunctional dGTP-pyrophosphohydrolase/thiamine phosphate synthase	Q7NS16	CV_3611
Signal transduction				
**Two-component system**				
	pilR	Two-component system, regulatory protein	Q7P1Y9	CV_0073
	qseB	Transcriptional regulatory protein qseB	Q7NZN1	CV_0891
	glnG	Nitrogen assimilation regulatory response regulator/transcription regulator protein	Q7NS35	CV_3592
	evgS	Sensor protein evgS	Q7NVE3	CV_2400
**Signal transduction mechanisms**				
	barA	Probable sensor/response regulator hybrid	Q7NSC3	CV_3503
	cheY	probable response regulator	Q7P1N2	CV_0181
Membrane transporte				
**ABC transportes**				
	gltJ	Glutamate/aspartate transport system permease	Q7P1U3	CV_0119
	CV_3922	probable transcription regulator, TetR family	Q7NR62	CV_3922
	LolD	Lipoprotein-releasing system ATP-binding protein	Q7NTU0	CV_2963
	hbpA	Heme-binding protein A	Q7NWK8	CV_1978
	mglA	Sugar ABC transporter, ATP-binding protein	Q7NVJ4	CV_2348
	CV_2982	probable amino-acid transport system permease ABC transporter protein	Q7NTS1	CV_2982
	rbsA	Ribose import ATP-binding protein RbsA	Q7NTN6	CV_3018
	dppA	probable binding protein component of ABC dipeptide transporter	Q7NR48	CV_3936
**Pilus biogenesis**				
	CV_3540	Probable fimbrial biogenesis and twitching motility protein	Q7NS86	CV_3540
	pilO	Type 4 fimbrial biogenesis protein PilO	Q7NZU0	CV_0831
	pilN	Type 4 fimbrial biogenesis protein PilN	Q7NZT9	CV_0832
**Bacterial secretion system**				
	hylA	probable hemolysin	Q7NWR2	CV_1918
**membrane protein**				
	lolB	Outer-membrane lipoprotein lolB	Q7NQS7	CV_4060
	wcaI	Probable glycosyl transferase	Q7NQW5	CV_4021
	yehX	probable ABC transporter ATP-binding protein	Q7NPU8	CV_4394
General Metabolism Amino acid				
**Metabolism Amino acid**	CV_0170	probable methyl transferase	Q7P1P3	CV_0170
	mdmC	O-methyltransferase	Q7P1M0	CV_0193
	CV_0785	methionine biosynthesis protein MetW	Q7NZY4	CV_0785

	hipO	Hippurate hydrolase hipO	Q7NWU5	CV_1884
	pabB	Anthranilate synthase	Q7NSL1	CV_3411
	hisH	Imidazole glycerol phosphate synthase subunit hisH	Q7P0F1	CV_0616
	astE	Succinylglutamate desuccinylase	Q7NU26	CV_2877
	ddlA	D-alanine--D-alanine ligase A ddlA	Q7NV72	CV_2472
	leuC	3-isopropylmalate dehydratase large subunit	Q7NUB6	CV_2784
	CV_0178	alanine racemase domain containing protein	Q7P1N5	CV_0178
Metabolism of other amino acids				
**Glutathione metabolism,**				
	gst	Probable glutathione S-transferase	Q7NZ36	CV_1086
	gst3	Glutathione S-transferase family protein	Q7NX54	CV_1775
	kdsA	2-dehydro-3-deoxyphosphooctonate aldolase	Q7NUF4	CV_2745
Translation				
**Ribosome**	*rplS*	50S ribosomal protein L19	Q7NRV7	CV_3672
	rpmB	50S ribosomal protein L28	Q7NSH0	CV_3456
	rimO	Ribosomal protein S12 methylthiotransferase RimO	Q7NYA1	CV_1373
	entE	2,3-dihydroxybenzoate-AMP ligase	Q7NXZ2	CV_1484
	CV_0256	tRNA-binding protein	Q7P1F7	CV_0256
Transcription				
**transcription regulation**	hydG	Two-component response sensor-transcriptional regulatory protein	Q7NZM5	CV_0897
	CV_1302	Probable bacterial regulatory helix-turn-helix proteins, araC family	Q7NYH2	CV_1302
	CV_3425	putative transcriptional regulatory protein	Q7NSJ9	CV_3425
	CV_2699	probable transcriptional regulator, TetR family	Q7NUJ8	CV_2699
	fnr	probable transcriptional regulator, Crp/Fnr family	Q7MBF0	CV_2708
	CV_3160	Bacterial regulatory protein, MarR	Q7NT99	CV_3160
	CV_4141	Probable AraC-family transcriptional regulator	Q7NQJ6	CV_4141
	rpoH	RNA polymerase sigma factor	Q7NQD4	CV_4206
	rpoS	RNA polymerase sigma factor	Q7NRU8	CV_3682
	CV_0539	Probable transcriptional regulator, MarR-family	Q7P0M7	CV_0539
	emrR	Transcriptional repressor emr operon, MarR family	Q7P000	CV_0769
	zntR	probable transcriptional regulator, MerR family	Q7NYW9	CV_1153
	CV_1779	probable transcriptional regulator, MerR family	Q7NX50	CV_1779
	yigN	probable periplasmic protein	Q7NV71	CV_2473
	ycjC	probable transcriptional regulator	Q7NWG8	CV_2021
Lipid metabolism				
**Metabolism Fatty Acid**	*cfa*	Cyclopropane-fatty-acyl-phospholipid synthase	Q7NZE4	CV_0978

	lpxH	UDP-2,3-diacylglucosamine hydrolase	Q7NT75	CV_3186
	paaG	probable enoyl-CoA hydratase	Q7NX66	CV_1763
	paaG	Enoyl-CoA hydratase	Q7NWA8	CV_2082
	ydiD	probable long chain fatty-acid CoA ligase	Q7NX49	CV_1780
	ugpQ	Glycerophosphodiester phosphodiesterase	Q7NRX6	CV_3652
	lipB	Octanoyltransferase	Q7NTG0	CV_3096
Peptidoglycan				
**Peptidoglycan**	dacB	Serine-type D-Ala-D-Ala carboxypeptidase	Q7NYZ7	CV_1125
Replication and DNA Repair				
	CV_1144	DinB family protein	Q7NYX8	CV_1144
**Base excision repair**				
	nth	Endonuclease III	Q7NSX6	CV_3293
	mutM	Formamidopyrimidine-DNA glycosylase	Q7NQS5	CV_4062
	ung	Uracil-DNA glycosylase	Q7P1I1	CV_0232
Cell motility				
**Flagellar Assembly**				
	flgA	probable flagella basal body P-ring formation protein flgA	Q7NZC3	CV_0999
	fliC2	Flagellin	Q7NV50	CV_2495
	flhF	Flagellar biosynthetic protein FlhF	Q7NZ98	CV_1024
**Bacterial chemotaxis**				
	CV_0257	probable methyl-accepting chemotaxis protein	Q7P1F6	CV_0257
	trg	probable methyl-accepting chemotaxis protein	Q7NYE6	CV_1328
	CV_1698	probable methyl-accepting chemotaxis protein	Q7NXC9	CV_1698
	CV_4155	probable methyl-accepting chemotaxis transducer	Q7NQI2	CV_4155
	trg	probable methyl-accepting chemotaxis protein	Q7NU51	CV_2852
	tar	probable methyl-accepting chemotaxis protein with HAMP domain	Q7NRX9	CV_3649
Nucleotide metabolism				
**Purine metabolism**	dgt	dGTPase	Q7MBE5	CV_3210
	purN	Phosphoribosylglycinamide formyltransferase	Q7NS11	CV_3616
	relA	GTP diphosphokinase	Q7NRS8	CV_3702
**Pyrimidine metabolism**				
	udk	Uridine kinase	Q7NZ37	CV_1085
Folding, soring and degradation				
**RNA degradation**	orn	Oligoribonuclease	Q7NVH5	CV_2368
**chaperone**				
	CV_4000	probable competence protein ComM	Q7NQY5	CV_4000
**Haemin-degrading**				
	CV_3897	Probable hemin degrading factor	Q7NR87	CV_3897
**proteolysis**				

	colA	probable microbial collagenase	Q7NWI8	CV_2001
	pepP	Xaa-Pro aminopeptidase	Q7NTD6	CV_3122
	pcp	Pyrrolidone-carboxylate peptidase	Q7NT84	CV_3176
	CV_3452	Peptidase M22 glycoprotease	Q7NSH4	CV_3452
	ptrB	Prolyl oligopeptidase family protein	Q7NSC4	CV_3502
CRISPR-associated protein				
	CV_1751	CRISPR-associated Csy4 family protein	Q7NX77	CV_1751
	CV_1754	CRISPR-associated protein, Csy1 family	Q7NX74	CV_1754
Cell cycle				
	CV_3387	segregation and condensation protein B	Q7NSN3	CV_3387
	CV_3543	sporulation domain containing protein	Q7NS83	CV_3543
	ftsJ	cell division protein FtsJ	Q7NRI3	CV3798
Acetylation				
**Histone deacetylase superfamily**	hda	Histone deacetylase	Q7NRU4	CV_3686
**GCN5-related N-acetyltransferase, Acyl-CoA N-acyltransferase**				
	CV_0453	probable acetyl transferase	Q7P0W2	CV_0453
	elaA	Probable ElaA protein	Q7P1Y6	CV_0076
	CV_3388	hypothetical acetyltransferase	Q7NSN2	CV_3388
	CV_3155	diamine N-acetyltransferase	Q7NTA4	CV_3155
OTHERS				
	T9J2	Latex allergen	Q7P0X4	CV_0441
	mpl	UDP-N-acetylmuramate: L-alanyl-gamma-D-glutamyl-meso-diaminopimelate ligase	Q7NXG0	CV_1666
	CV_2336	probable ThiJ/PfpI family protein	Q7NVK6	CV_2336
	CV_2923	probable partition-related protein	Q7NTY0	CV_2923
	hyuA	Hydantoin racemase	Q7NTB3	CV_3146
	CV_3201	thioesterase superfamily protein	Q7NT60	CV_3201
	CV_3236	ThiJ/PfpI domain-containing protein	Q7NT28	CV_3236
	hflX	GTP-binding protein hflX hflX	Q7NS94	CV_3532
	pcm	Protein-L-isoaspartate O-methyltransferase	Q7NRV0	CV_3680
	phuR	Probable TonB-dependent receptor	Q7NR88	CV_3896
	CV_0565	Hemerythrin HHE cation binding domain protein	Q7P0K2	CV_0565
	CV_2075	molybdopterin binding domain containing protein	Q7NWB5	CV_2075
	anmK	Anhydro-N-acetylmuramic acid kinase	Q7NV67	CV_2478
	CV_0156	adenylate cyclase	Q7P1Q7	CV_0156
	CV_0212	esterase YqiA	Q7P1K1	CV_0212
	CV_0791	ybaK/ebsC protein	Q7NZX8	CV_0791
	CV_2925	exopolyphosphatase-like protein	Q7NTX8	CV_2925
	CV_3157	kinase inhibitor	Q7NTA2	CV_3157
**Ubiquinone and other terpenoid-quinone biosynthesis,**				

	ubiF	Monooxygenase	Q7NYZ0	CV_1132
	entC	Isochorismate synthase EntC / MenF	Q7NXZ1	CV_1485
**YD repeat,**				
	rhsC	Probable rhs-related protein	Q7NYN5	CV_1238
Viral				
	CV_1315	probable phage-related protein	Q7NYF9	CV_1315
	ycfE	probable bacteriophge tail fiber protein	Q7P176	CV_0337
	CV_0339	probable bacteriophge tail fiber protein	Q7P174	CV_0339
	CV_0338	Putative uncharacterized protein	Q7P175	CV_0338
	CV_0347	Probable phage virion	Q7P167	CV_0347
	CV_0343	Probable phage FluMu protein gp47	Q7P170	CV_0343
	ydfM	probable tail fiber assembly protein	Q7P173	CV_0340
	CV_0345	probable bacteriophage base plate protein	Q7P169	CV_0345
	CV_0346	probable bacteriophage tail protein	Q7P168	CV_0346
	CV_0348	peptidoglycan-binding domain 1 protein	Q7P166	CV_0348
	CV_0342	phage tail protein	Q7P171	CV_0342
	CV_0407	phage protein	Q7P107	CV_0407
	CV_0418	Probable tail fiber-related protein	Q7P0Z7	CV_0418
	CV_0417	Probable phage baseplate component	Q7P0Z8	CV_0417
	CV_0419	probable tail fiber assembly-like protein	Q7P0Z6	CV_0419
	CV_0422	probable bacteriophage baseplate assembly protein	Q7P0Z3	CV_0422
	CV_0429	probable bacteriophage tail fiber protein	Q7P0Y6	CV_0429
	CV_0424	putative pyocin R2_PP, tail fiber protein	Q7P0Z1	CV_0424
	CV_0425	Putative uncharacterized protein	Q7P0Z0	CV_0425
	CV_0431	probable bacteriophage protein	Q7P0Y4	CV_0431
	CV_0432	probable tail fiber assembly protein	Q7P0Y3	CV_0432
Putative uncharacterized protein	CV_4246	Putative uncharacterized protein	Q7NQ94	CV_4246
	CV_0011	Putative uncharacterized protein	Q7P250	CV_0011
	CV_2113	Putative uncharacterized protein	Q7NW77	CV_2113
	CV_0053	Putative uncharacterized protein	Q7P208	CV_0053
	CV_3151	Putative uncharacterized protein	Q7NTA8	CV_3151
	CV_3265	Putative uncharacterized protein	Q7NT00	CV_3265
	CV_3475	Putative uncharacterized protein	Q7NSF1	CV_3475
	CV_1311	Putative uncharacterized protein	Q7NYG3	CV_1311
	CV_4296	Putative uncharacterized protein	Q7NQ44	CV_4296
	CV_3599	Putative uncharacterized protein	Q7NS28	CV_3599
	CV_3581	Cupin 4 family protein	Q7NS46	CV_3581
	CV_0746	Putative uncharacterized protein	Q7P023	CV_0746
	CV_2902	Putative uncharacterized protein	Q7NU01	CV_2902
	CV_2971	Putative uncharacterized protein	Q7NTT2	CV_2971
	CV_3623	Putative uncharacterized protein	Q7NS04	CV_3623
	CV_0330	Putative uncharacterized protein	Q7P183	CV_0330

Supplementary Table S5: Protein Functional Category Classification, uniquely detected in the control condition.

Pathway classification				
Biosynthese of secondary metabolites	Gene name	Description	Uniprot accession	ORF
**Terpenoid backbone biosynthesis**	ispD	2-C-methyl-D-erythritol 4-phosphate cytidylyltransferase	Q7NYL6	CV_1258
	uppS	Undecaprenyl pyrophosphate synthase	Q7NVZ0	CV_2200
**Streptomycin biosynthesis, Inositol phosphate metabolism**				
	suhB2	Myo-inositol-1(Or 4)-monophosphatase	Q7P148	CV_0366
Translation				
**Ribosome**	rpsI	30S ribosomal protein S9	Q7NRT4	CV_3696
	rplK	50S ribosomal protein L11	P60100	CV_4197
	rplU	50S ribosomal protein L21	Q7NZS3	CV_0848
**Aminoacyl-tRNA byosynthesis**				
	CV_2329	tRNA-dihydrouridine synthase	Q7NVL3	CV_2329
	rluB	Pseudouridylate synthase	Q7NQJ1	CV_4146
	truA	tRNA pseudouridine synthase A	Q7NUD6	CV_2764
	trmD	tRNA (guanine-N(1)-)-methyltransferase	Q7MBD6	CV_3673
Amino acid metabolism				
**Amino acid metabolism**				
	dadB	Alanine racemase 2	Q7NRI8	CV_3793
	trpD	Anthranilate phosphoribosyltransferase	Q7NW17	CV_2173
	cysE	Serine O-acetyltransferase	Q7P217	CV_0044
	CV_0106	probable O-methyltransferase	Q7P1V6	CV_0106
	dapC	Succinyldiaminopimelate transaminase	Q7P0W4	CV_0451
	CV_0748	probable transformylase	Q7P021	CV_0748
	murI	Glutamate racemase	Q7NYS0	*CV_1203*

	CV_3407	probable prephenate dehydrogenase	Q7NSL5	CV_3407
	CV_3753	probable alanyl-tRNA synthetase related protein	Q7NRM7	CV_3753
	glsA	Glutaminase	Q7NQH9	CV_4158
Metabolism nucleic acid				
**Purine metabolism**				
	apaH	Bis(5'-nucleosyl)-tetraphosphatase, symmetrical apaH	Q7NQS0	CV_4067
	CV_0033	probable ribose-phosphate diphosphokinase	Q7P228	CV_0033
	guaC	GMP reductase guaC	Q7NTY1	CV_4158
	CV_3720	hydrolase, TatD family	Q7NRR0	CV_3720
	rluD	Pseudouridine synthase	Q7NVZ8	CV_2192
**DNA metabolic process**				
	CV_2171	probable adenine-specific methylase	Q7NW19	CV_2171
Replication and repair				
	CV_2089	probable ATP-dependent RNA helicase	Q7NWA1	CV_2089
	dnaQ	DNA-directed DNA polymerase, epsilon chain	Q7NYL7	CV_1257
	recJ	Exodeoxyribonuclease VII	Q7NV69	CV_2475
	CV_3454	Probable DNA-directed DNA polymerase, bacteriophage-type	Q7NSH2	CV_3454
	recO	DNA repair protein recO	Q7NWC1	CV_1612
	radA	DNA repair protein radA	Q7NTS9	CV_2974
	dnaG	DNA primase	Q7NRL7	CV_3763
	recB	Exodeoxyribonuclease V, beta subunit	Q7NQR1	CV_4076
Folding, sorting and degradation				
**Proteolysis**				
	CV_0153	peptidase M48, Ste24p	Q7P1R0	CV_0153
	ydcP	probable collagenase	Q7P1E7	*CV_0266*
	CV_1270	probable dipeptidyl anminopeptidase	Q7NYK4	CV_1270
	CV_1999	20S proteasome A and B subunits	Q7NWJ0	CV_1999

**Hydrolisis**				
	CV_1727	Cof-like hydrolase	Q7NXA0	CV_1727
Cell envelope biogenesis, outer membrane,				
**Membrane protein**				
	CV_0228	probable beta-1,4- glucosyltransferase	Q7P1I5	CV_0228
	mltA	Peptidoglycan N-acetylmuramoylhydrolase	Q7NRM1	CV_3759
**Lipoprotein**				
	vacJ	Lipoprotein	Q7P0X1	CV_0444
	CV_1235	probable lipoprotein	Q7NYN8	CV_1235
Transcription regulator activity				
	CV_0635	putative PAS/PAC sensor protein	Q7P0D2	CV_0635
	CV_1077	probable transcriptional regulator, TetR family	Q7NZ45	CV_1077
	CV_2059	anti sigma-E protein, RseA	Q7NWD1	CV_2059
	CV_2323	probable transcriptional regulatory protein, LuxR family	Q7NVL9	CV_2323
	CV_2476	probable signal peptide protein	Q7NV68	CV_2476
	CV_3008	response regulator receiver protein	Q7NTP6	CV_3008
	CV_3051	probable transcriptional regulator lacI family	Q7NTK3	CV_3051
	CV_3919	probable transcriptional regulator, AsnC family	Q7NR65	CV_3919
Signal transduction				
**Two-component system**				
	tap	Methyl-accepting chemotaxis protein IV	Q7P0E0	CV_0627
	glnD	[Protein-PII] uridylyltransferase	Q7NTY6	CV_2917
	yfhA	probable two-component transcriptional regulator	Q7NQC0	*CV_4220*
	hprK	HPr kinase/phosphorylase	Q7NST5	CV_3335
Cell motility				
**Bacterial chemotaxis**				
	tsr	probable methyl-accepting chemotaxis transducer	Q7P055	CV_0713

	motB2	Chemotaxis motB protein	Q7NWG2	CV_2027
**Flagellar assembly**				
	fliD	Flagellar hook-associated protein	Q7NTR0	CV_2994
	fliG2	Flagellar motor switch protein	Q7NTQ6	CV_2998
	fliN	probable flagellar motor switch protein	Q7NTQ2	*CV_3002*
	flgI1	Flagellar P-ring protein 1	Q7NXB9	*CV_2881*
Carbohydrate metabolism				
**Glyoxylate and dicarboxylate metabolism**				
	CV_1622	probable formate dehydrogenase	Q7NXK4	CV_1622
**Amino sugar and nucleotide sugar metabolism**				
	CV_0818	probable glycosyltransferase	Q7NZV3	CV_0818
	rfbU	Lipopolysaccharide biosynthesis protein	Q7NZU7	CV_0824
	CV_1007	serine/threonine protein kinase	Q7NZB5	CV_1007
	CV_1160	probable glycosyl transferase	Q7NYW2	CV_1160
	CV_1493	probable b-N-acetylhexosaminidase	Q7NXY3	CV_1493
	cpbD	Carbohydrate-binding protein	Q7NUV5	CV_2592
	fruK	1-phosphofructokinase	Q7NTK1	CV_3053
	fruA	Protein-N p-phosphohistidine-sugar phosphotransferase	Q7NTK0	CV_3054
	galE	UDP-glucose 4-epimerase	Q7NRA0	CV_3884
	rfbG	CDPglucose 4,6-dehydratase	Q7NR92	CV_3892
	pmrF	probable dolichyl-phosphate b-D-mannosyltransferase	Q7P020	*CV_0749*
Membrane transport				
**ABC transporters,**				
	dppB	Dipeptide transport system permease protein	Q7NZ24	CV_1098
	CV_1560	probable iron transport system ATP-binding protein	Q7NXR6	CV_1560
	exbB1	Biopolymer transport protein	Q7M7G2	CV_1972

	CV_2295	probable ABC transporter system, ATP-binding protein	Q7NVP7	CV_2295
	CV_2915	permease, YjgP/YjgQ family	Q7NTY8	CV_2915
	CV_3734	probable ATP-binding component, ABC transporter	Q7NRP6	CV_3734
	CV_1681	probable ABC transporter, periplasmic binding protein	Q7NXE6	CV_1681
	CV_0447	probable ABC transport system substrate-binding protein	Q7P0W8	CV_0447
Metabolism of cofactors na vitamins				
**Riboflavin metabolism**				
	ribD	Diaminohydroxyphosphoribosylaminopyrimidine deaminase ribD	Q7NYI4	CV_1290
**vitamin metabolic process**				
	cobC	Cobalamin biosynthetic protein	Q7NXQ2	CV_1574
	CV_3067	probable cobalamin synthesis protein	Q7NTI7	CV_3067
**Folate biosynthesis**				
	folP	Dihydropteroate synthase	Q7NRI5	CV_3054
**Biotin metabolism**				
	birA	Biotin acetyl-CoA-carboxylase synthetase	Q7P0T0	CV_0486
	bioC	Biotin synthesis protein	Q7NPW6	CV_4376
	bioB	Biotin synthase	Q7NPW1	CV_4381
CRISPR-associated protein Csy2				
	CV_1753	CRISPR-associated protein, Csy2 family	Q7NX75	CV_1753
Fatty acid metabolism				
	rfaF	ADP-heptose--LPS heptosyltransferase II	Q7NW98	CV_2092
	paaF	probable enoyl-CoA hydratase	Q7NV60	*CV_2485*
	plsX	fatty acid/phospholipid biosynthesis protein	Q7NSK6	CV_3417
	lpxB	Lipid-A-disaccharide synthase	Q7NVY1	CV_2209

	CV_4051	probable long chain fatty-acid CoA ligase	Q7NQT6	CV_4051
Cell division				
	ftsK	Cell division ftsk transmembrane protein	Q7NV66	CV_2479
Iron-sulphur cluster binding protein				
	ykgF	Probable iron-sulphur protein	Q7NTM6	CV_3028
	CV_3886	Probable Fe-S oxidoreductase	Q7NR98	CV_3886
Energy metabolism				
**Cytochrome c, class I**				
	CV_3056	probable cytochrome c precursor	Q7NTJ8	CV_3056
**Other**				
	CV_2363	GAF domain-containing protein	Q7NVI0	CV_2363
	CV_2978	probable geranylgeranyl-diphosphate geranylgeranyltransferase	Q7NTS5	CV_2978
	nfsA	Oxygen-insensitive NADPH nitroreductase	Q7NSC6	CV_3500
	CV_3724	aminodeoxychorismate lyase	Q7NRQ6	CV_3724
	CV_3822	probable N-acetylmuramoyl-L-alanine amidase	Q7NRF9	CV_3822
	rsgA2	Putative ribosome biogenesis GTPase RsgA 2	Q7NVH8	CV_2365
	CV_3888	probable methyltransferase	Q7NR96	CV_3888
	moeB	Molybdopterin biosynthesis MoeB protein	Q7NQ82	CV_4258
	phoD	Phosphodiesterase I	Q7NQ58	CV_4282
	mreC	Rod shape-determining protein mreC	Q7NPY4	CV_4358
	mrdA	Penicillin-binding protein 2	Q7NPY2	CV_4360
	ppnK	Probable inorganic polyphosphate/ATP-NAD kinase	Q7NVM0	CV_2322
	queC	7-cyano-7-deazaguanine synthase	Q7NSB9	CV_3507
	CV_4092	probable aldehyde dehydrogenase	Q7NQP5	CV_4092
Putative uncharacterized protein				
	CV_1445	Putative uncharacterized protein	Q7NY30	CV_1445
	CV_3513	UPF0276 protein	Q7NSB3	CV_3513

	CV_1711	Putative uncharacterized protein	Q7NXB6	CV_1711
	CV_2561	Putative uncharacterized protein	Q7NUY6	CV_2561
	CV_3785	Putative uncharacterized protein	Q7NRJ6	CV_3785
	CV_1784	Putative uncharacterized protein	Q7NX45	CV_1784
	CV_0286	Putative uncharacterized protein	Q7P1C7	CV_0286
	CV_3423	Putative uncharacterized protein	Q7NSK1	CV_3423
	CV_2149	Putative uncharacterized protein	Q7NW41	CV_2149
	CV_0913	Putative uncharacterized protein	Q7NZK9	CV_0913
	CV_3974	Putative uncharacterized protein	Q7NR11	CV_3974
	CV_2003	Putative uncharacterized protein	Q7NWI6	CV_2003
	CV_0373	Putative uncharacterized protein	Q7P141	CV_0373
	CV_2890	Putative uncharacterized protein	Q7NU13	CV_2890
	CV_2799	Putative uncharacterized protein	Q7NUA4	CV_2799
	CV_3937	Putative uncharacterized protein	Q7NR47	CV_3937
	CV_4122	Putative uncharacterized protein	Q7NQL5	CV_4122
	CV_2486	Conserved hypothetical protein	Q7NV59	CV_2486
	CV_4304	Putative uncharacterized protein	Q7NQ36	CV_4304

Supplementary Table S6: List of the primers used in this study.

Primer name	Sequence
CV3027 Forward	AGCACCAGTCTGTTGATCGAG
CV3027 Reverse	CTGGATACTTGCGGCTGATAA
CV4376 Forward	AGATCGCGTTGACCTACGAC
CV4376 Reverse	TAGCGCTCGTACGCTTCTTC
16s Forward	TGTAGCAGTGAAATGCGTAGAGATG
16s Reverse	CCACGCTTTCGTGCATGAG
